# DNA Binding Study of a Redox Active Enantiopure Bis(arylimino)acenaphthene (BIAN) Os(II) Bipyridine Complex

**DOI:** 10.1002/cbic.202500536

**Published:** 2025-10-28

**Authors:** Judit Fodor, Susan J. Quinn, Andrew D. Phillips

**Affiliations:** ^1^ School of Chemistry University College Dublin Dublin 4 D04 N2E5 Ireland

**Keywords:** bis(arylimino)acenaphthene, bioinorganic, DNA binding, osmium, redox‐active

## Abstract

This work presents the synthesis, characterization, and evaluation of DNA binding for a bis(arylimino)acenaphthene (BIAN) containing Os(II) octahedral dipyridyl complex [Os(dmbpy)_2_BIAN](An)_2_ [**1**](An)_2_ (dmbpy = 4,4′‐dimethyl‐2,2′‐bipyridyl, BIAN = bis(arylimino)acenaphthene, An = PF_6_^−^ or Cl^−^). Complex **1** is characterized by X‐ray crystallography and cyclic voltammetry, which reveal a series of fully reversible redox couples. The visible absorption spectrum is dominated by metal‐to‐ligand charge‐transfer bands between 400 and 600 nm, whose assignment is supported by time dependent density functional theory computational studies with solvent corrections. For the first time, chiral discriminating chromatography is used to resolve the Δ‐ and Λ‐enantiomers of a metal BIAN complex. UV–visible absorption and circular dichroism measurements reveal significant differences in the binding strength of the enantiomers to double‐stranded natural DNA, with a greater affinity observed for Δ‐**1** (*K*_b_ ≈ 10^6 ^ M^−1^), which is comparable to that observed for other group 8 polypyridyl intercalating species and is supported by the thermal denaturation temperature recorded in the presence of DNA. Linear dichroism confirms that both enantiomeric species bind through the intercalation of the BIAN component. The development of DNA‐binding chiral metal complexes containing redox‐active ligands described in this work offers the potential to expand the activity of therapeutic bioinorganic systems in new directions.

## Graphical abstract

The synthesis of a novel dicationic osmium polypyridyl complex featuring the bis(arylimino)acenaphthene ligand demonstrates interesting redox properties. Successful enantiomer resolution reveals that the delta isomer is a strong DNA grove binder.

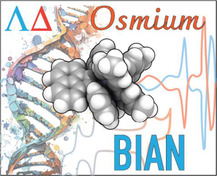

## 1 Introduction

Group 8 octahedral d^6^‐ruthenium(II) and ‐osmium(II) polypyridyl complexes consisting of 2,2′‐bipyridine or 1,10‐phenanthroline ligands are considered classic chromophores^[^ ^1^ ^]^ and have been exploited in a highly diverse range of photochemical‐ and redox‐based^[^ ^2^ ^]^ (water oxidation)^[^ ^3^ ^]^ applications extending from photocatalysts and solar photon energy conversion as the basis of dye‐sensitized solar cells,^[^ ^4^ ^,^ ^5^ ^]^ to diagnostic and therapeutic applications.^[^ ^6^ ^–^ ^8^ ^]^ These complexes are characterized by strong visible absorptions arising from facile metal‐to‐ligand charge‐transfer (MLCT) transitions, where the ubiquitous [Ru(bpy)_3_]^2+^ ion features a strong ^1^MLCT band near 450 nm (with higher‐energy *π*–π* bands around 300 nm),^[^ ^9^ ^,^ ^10^ ^]^ also observed for the [Os(bpy)_3_]^2+^ species. However, in contrast to the [Ru(bpy)_3_]^2+^ species, the Os(II) complex also features less‐intense broad bands ranging from 520 to 700 nm due to formally spin‐forbidden (triplet) ^3^MLCT absorptions.^[^ ^11^ ^]^ The corresponding osmium‐centered polypyridyl complexes have generally been less studied than the Ru counterparts,^[^ ^12^ ^]^ but nevertheless, feature interesting and tuneable photochemical properties.^[^ ^12^ ^]^ One interesting feature of heavier Os centers is the significantly stronger spin–orbit coupling (SOC) due to relativistic effects experienced by the 5d orbitals, enabling the observation of nominally spin‐forbidden processes, such as S^0^ → ^3^MLCT excitation in the red–near infrared (NIR) region,^[^ ^10^ ^]^ which are absent in the corresponding Ru(II) complexes. After photoexcitation, the initial singlet MLCT state rapidly undergoes intersystem crossing (aided by metal SOC) to a triplet (^3^MLCT) excited.^[^ ^13^ ^]^ In the case of Ru(II) complexes, long‐lived phosphorescent ^3^MLCT states are commonly observed to survive well into the hundreds of nanoseconds or microseconds, whereas the analogous Os(II) complexes typically relax several orders of magnitude faster with <1% emission yields.^[^ ^11^ ^]^

In parallel with the development of the photochemistry of ruthenium(II) and ‐osmium(II) polypyridyl complexes, biological applications have surged, not only due to phosphorescent properties and ability to sensitize singlet oxygen formation,^[^ ^14^ ^]^ but in part to the high kinetic stability of these complexes against ligand dissociation. In particular, the DNA binding interactions of transition metal complexes containing polypyridyl ligands capable of strong intercalative binding to DNA have been extensively reported.^[^ ^15^, ^16^, ^17^, ^18^ ^–^ ^19^ ^]^ In these studies, the inclusion of the extended dppz (dipyrido[3,2‐*a*:2′,3′‐*c*]phenazine) intercalating ligand and its derivatives has been widely exploited.^[^ ^19^, ^20^ ^–^ ^22^ ^]^ The biological application of osmium polypyridyl complexes has been the subject of numerous studies,^[^ ^23^, ^24^, ^25^, ^26^ ^–^ ^30^ ^]^ which included recent imaging studies that exploited their NIR luminescence and the high atomic mass for confocal microscopy^[^ ^27^ ^,^ ^28^ ^]^ and transmission electron microscopy^[^ ^29^ ^]^ resolution of osmium complex uptake in cells. Importantly, the dynamic resolution of Δ‐ and Λ‐enantiomers of polypyridyl complexes provides an additional avenue to modulate DNA binding as the enantiomers bind differently to the chiral DNA structure.^[^ ^31^, ^32^ ^–^ ^33^ ^]^ For example, the binding and luminescent response of dppz‐containing osmium polypyridyl complexes to both double‐stranded (B‐DNA) and quadruplex DNA has been shown to be sensitive to the enantiomeric form about the metal centre.^[^ ^21^ ^,^ ^34^ ^]^

Although a plethora of Ru(II)‐ and Os(II)‐polypyridyl complexes comprising either 2,2‐bipyridyl or extensively modified dipyridyl ligands are known, only a few studies have explored other potential classes of chelating N,N‐ligands incorporating *π*‐bonds. These include benzimidazoles^[^ ^35^ ^]^ and bis‐2‐oxazolines.^[^ ^36^ ^]^ The scope of *α*,*α*‐diimine ligands has expanded over the past decades to include a more diverse range of backbone components, including bis(arylimino)acenaphthane (BIAN).^[^ ^37^ ^]^ Brookhart and others have pioneered this ligand class for precise catalytic control of various cationic‐based polymerization reactions,^[^ ^38^ ^]^ as the electronic and steric properties of BIANs are readily tuned through modification of the flanking N‐aryl groups. BIANs are considerably stronger *σ*‐electron donors than the acyclic *α*,*α*‐diimine counterparts, but the strong *π*‐electron accepting nature of the naphthalene backbone enables the stabilization of a wide range of species bearing high‐ and low‐oxidation state metals,^[^ ^39^ ^]^ and in comparison, the gas phase DFT calculated highest occupied molecular orbital to lowest unoccupied molecular orbital gap of Phenyl‐BIAN (2.22 eV) is significantly smaller than that of 2,2‐bipyridyl ligands (3.28 eV).^[^ ^40^ ^]^ More recent studies have indicated that coupling between the metal‐diimine and naphthalene components enables interesting redox properties through an efficient *π*‐delocalization of unpaired electrons, affording otherwise unstable radical and dianionic species.^[^ ^6^ ^]^

The development of metal complexes containing redox‐active ligands offers the potential to expand the activity of therapeutic bioinorganic systems. In this regard, BIAN ligands are particularly attractive due to their demonstrated ability to support consecutive oxidation states. Despite the extensive application of the BIAN ligand in catalysis, very few complexes have been prepared with the primary goal of biological activity. Examples include a series of vanadocene [Cp_2_V(IV)BIAN]OTf^[^ ^41^ ^]^ and Cu(II)‐BIAN complexes^[^ ^42^ ^]^ with potent anticancer properties. Calhorda et al. prepared a series of *η* ^3^‐allylic Mo(CO)_2_ complexes^[^ ^43^ ^]^ featuring a series of *para*‐substituted BIANs with ctDNA binding constants in the range of 7.50–0.22 × 10^6 ^M^−1^, while Pt(II)‐based BIAN/bipy, and Pd(II) BIAN/dmit^[^ ^44^ ^]^ (dmit = 1,3‐dithia‐2‐thione‐4,5‐dithiolate) and BIAN/dsit (dsit = 1,3‐dithia‐2‐thione‐4,5‐diselenolate) supported species demonstrated less effective DNA binding in the range of 6–10 × 10^4 ^M^−1^, but with the TUNEL assay^[^ ^45^ ^]^ demonstrated significant DNA fragmentation of the HT29 cancer cell line similar to that induced by cisplatin.^[^ ^45^ ^]^ In the case of the BIAN containing complexes experiments, have revealed significant generation of reactive oxygen species (ROS).^[^ ^44^ ^,^ ^45^ ^]^ Given the therapeutic potential of these complexes, we now report the synthesis, characterization, photochemical, and DNA intercalation properties of a new class of Os(II) 2,2‐dipyridyl complex featuring the BIAN ligand.

## 2 Results and Discussion

### 2.1 Synthesis and Enantiomeric Resolution

**Scheme 1:**
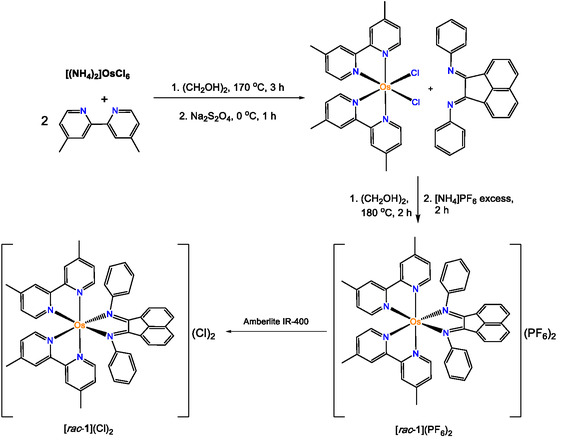
Synthetic scheme for [Os(dmbpy)_2_BIAN]^2+^ complexes [*rac*‐**1**](PF_6_)_2_/(Cl)_2_ and the precursor Os(dmbpy)_2_Cl_2_.

**Figure 1:**
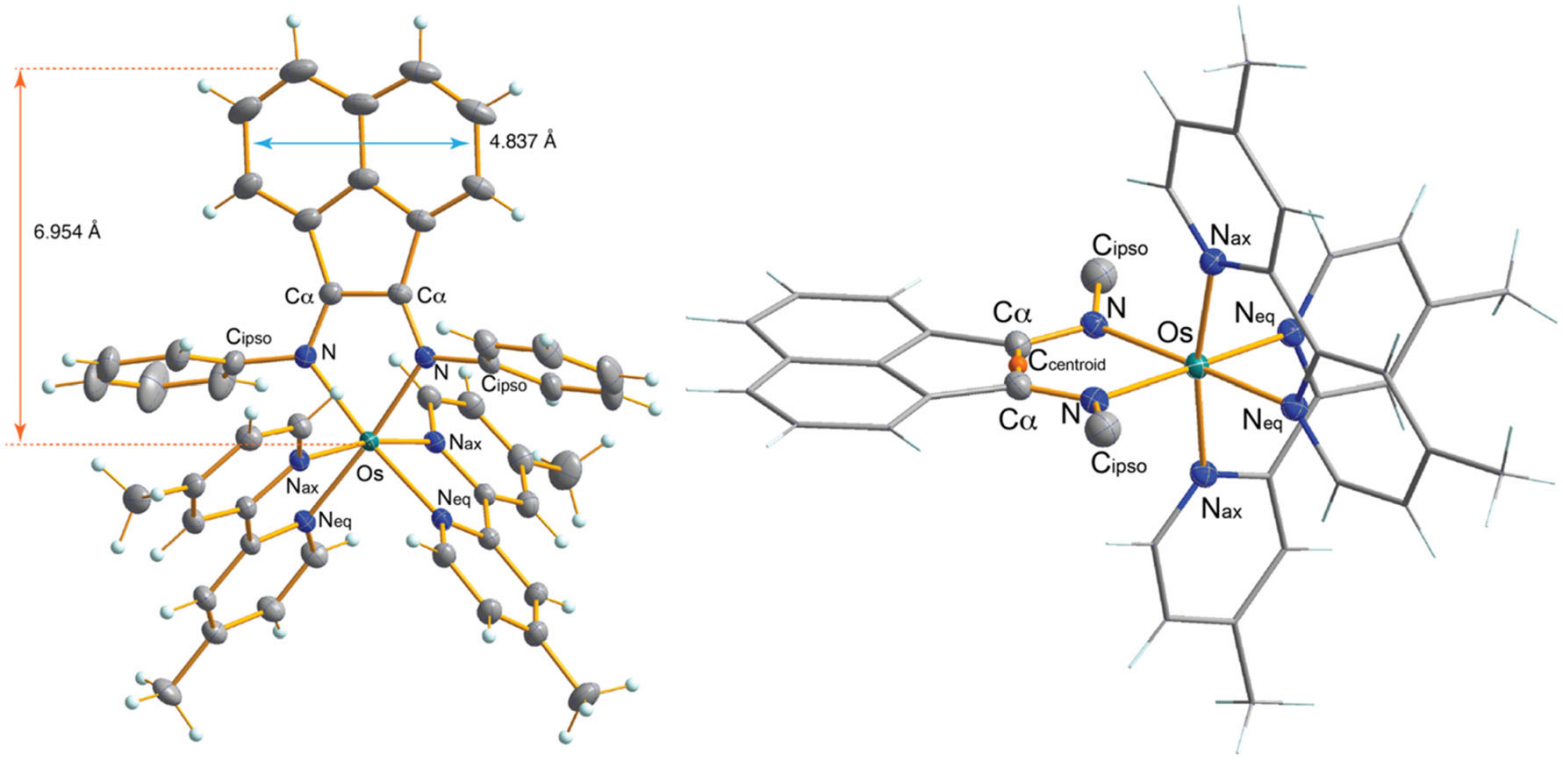
(Left) Solid‐state structure of the dicationic component of [Λ‐**1**](PF_6_)_2_ drawn with 30% probability ellipsoids. (Right) Side view of the dicationic component of [Λ‐**1**](PF_6_)_2_ with the flanking N‐aryl groups removed. Transverse (blue arrow) and sagittal (orange arrow) ligand distances are shown. For both figures, the PF_6_ counterions and DCM solvates have been omitted for clarity.

The synthesis of the dicationic octahedral [Os(dmbpy)_2_BIAN](An)_2_ [**1**](An)_2_ (dmbpy = 4,4′‐dimethyl‐2,2′‐bipyridyl, An = PF_6_^−^ or Cl^−^) is based upon modifications of standard protocols used to prepare a wide variety of N_6_‐hexacoordinate Os^2+^ species^[^ ^46^ ^]^ starting with Os(dmbpy)_2_Cl_2_ synthesized in a two‐step process involving reduction of Os^3+^ to Os^2+^ using sodium dithionite, **Scheme** **1** as described by Meyer et al.^[^ ^23^ ^]^ The N,N‐phenyl‐BIAN ligand was prepared using the ZnCl_2_ mediated template method according to previously reported procedures^[^ ^47^ ^]^ and was combined with the Os(dmbpy)_2_Cl_2_ precursor dissolved in dry and degassed ethylene glycol with heating at 180 °C in a pressure‐resistant glass vessel for 2 h. Subsequently, [*rac*‐**1**](PF_6_)_2_ was precipitated as a red powder by adding a concentrated aqueous solution of [NH_4_]PF_6_ and further purified on a silica column (CNCH_3_:H_2_O:NaNO_3_ (95:4:1)), affording a yield of 33% (Scheme 1). Product formation and purity were confirmed by ^1^H, ^13^C, ^19^F, ^31^P NMR, electrospray ionziation mass spectrometry, and elemental analysis (Figure S1‐2, Supporting Information). NMR conformation of BIAN binding indicates a typical deshielding of the protons associated with the naphthalene component. The complex [*rac*‐**1**](An)_2_ (An = PF_6_^−^) is soluble in a wide range of organic solvents, including MeCN, dichloromethane, and methanol, but is poorly soluble in water, whereas [*rac*‐**1**](Cl)_2_ demonstrates higher aqueous solubility. Finally, [Λ‐**1**](PF_6_)_2_ was preferentially crystalized from the racemic mixture and its structure confirmed by single crystal X‐ray crystallography (**Figure** **1**).

Chromatographic resolution of the Δ‐ and Λ‐enantiomers initially required counterion exchange from [*rac*‐**1**](PF_6_)_2_ to [*rac*–**1**](Cl)_2_ by stirring with Amberlite ion exchange resin, in methanol, Scheme 1. It was possible to chirally separate and resolve [*rac*–**1**](Cl)_2_ both SP and the CM Sephadex ion‐exchange stationary phases. However, SP media gave better separation of the bands in a shorter effective column length with more concentrated bands (Figure S3, Supporting Information). The aqueous solution of [*rac*–**1**](Cl)_2_ was loaded onto the Sephadex column in the eluent, sodium (−)–O, O–Dibenzoyl‐L‐tartaric acid buffer (0.1 M), following previous reported procedures.^[^ ^21^ ^,^ ^48^, ^49^ ^–^ ^50^ ^]^ The tartrate counterion was removed by ion exchange to reform the PF_6_^−^ salt, followed by filtration of the separated enantiomer dissolved in MeCN to remove excess salt. The [Δ‐**1**](Cl)_2_ enantiomer eluted first due to its higher affinity for the (−) tartrate salt, but this affinity also resulted in difficulty isolating the chloride salt of the complex and the removal of the tartrate salt. UV–visible absorption and circular dichroism (CD) spectroscopic measurements confirmed the high purity of the separated enantiomers, which had nearly identical absorption and CD spectra (Figure S3‐S4, Table S1, Supporting Information).^[^ ^51^ ^]^

### 2.2 Solid‐State Characterization

Suitable single crystals of [Λ‐**1**](PF_6_)_2_ were grown by slow diffusion of the antisolvent n‐pentane into a concentrated solution of the complex dissolved in DCM over several weeks. The unit cell contains two highly disordered PF_6_^−^ counterions and several DCM solvates. Selected metric parameters are given in **Table** **1**, (full parameters given in Tables S2–S7, Supporting Information). The dicationic structure of [Λ‐**1**](PF_6_)_2_ (Figure 1) features a typical axial distortion associated with octahedral group 8 complexes containing a [M(L)_2_]^+2^ fragment (L = bpy or phen), whereby the axial N centers of the dmbpy ligands are rotated by 99.5(1)° and 102.5(2)° from the BIAN ring plane as indicated by the N_ax_–Os–C_centroid_–C*α* dihedral angles (Figure 1). However, this axial distortion is greater than the 93.0(3)° found in [Os(bpy)_3_](PF_6_)_2_. The BIAN and dmbpy ligands all adopt similar chelation angles of 77.93(7)°, 77.38(7)°, and 77.74(7)°, respectively, significantly narrower than the 89.3(2)° N–Os–N in [Os(bpy)_3_](PF_6_)_2_.^[^ ^52^ ^]^ A second distortion of the Os octahedral geometry is the N_ax_–Os–N_ax_ bond angle (169.41(7)°) that deviates significantly from 180°, which is narrower than all the *trans* N–Os–N bond angles in [Os(bpy)_3_](PF_6_)_2_, being 172.4(2)°. The two flanking N‐phenyl groups of the coordinated BIAN are positioned orthogonal to the central ring plane with a slight forward tilt.^[^ ^53^ ^]^ As commonly observed for coordinated BIAN ligands, the central C_*α*_—C_*α*_ bond is significantly shortened (1.455(3) Å) compared to the nonligated species (1.526(2) Å),^[^ ^54^ ^]^ while the equivalent two imine bonds are lengthened (1.311(3) Å versus 1.275(2) Å). Barton et al., classified different types of metallo‐DNA binders based on the two dimensions of the external component of the interacting ligand, specifically, the transverse‐ligand distance and the sagittal length starting from the metal center to the maximum end of the ligand.^[^ ^55^ ^]^ Short ligand transverse and sagittal distances (5.1 Å) are associated with complexes that are DNA groove binders, whereas intermediate‐sized systems are metallointercalators (6.3 to 9.2 Å) and species with long ligand transverse distances (>11.3 Å) favor metallo‐DNA insertion. Measurement of the BIAN ligand in [Λ‐**1**](PF_6_)_2_ reveals a short transverse length of 4.84 Å and a sagittal distance of 6.95 Å, suggesting that this Os complex is a metallointercalator bordering on a groove binding behavior.

**Table 1:** Selected bond lengths (Å) and angles (°) for [Λ‐**1**](PF_6_)_2_.

Parameter	Bond lengths [Å]	Bond angles [°]	
Os–N (BIAN)	2.037(2) 2.047(2)	N–Os–N N_ax–_Os–N_eq_ N_ax_–Os–N_eq_	77.94(7) 77.38(7) 77.74(7)
Os–N_ax_ Os–N_eq_	2.048(2) 2.066(2) 2.070(2) 2.071(2)	Os–N–C_*α*_	115.49(13) 115.95(13)
BIAN C_*α*_– N BIAN C_*α*_– C_*α*_	1.312(3) 1.455(3)	N–C_*α*_–C_*α*_	114.90(17) 115.33(17)
BIAN N–C_ipso_	1.439(2) 1.436(3)	N_ax_–Os–N_ax_ N–Os–N_eq_ N–Os–N_eq_	169.40(6) 175.41(6) 174.23(6)

### 2.3 Electrochemical Studies

**Figure 2:**
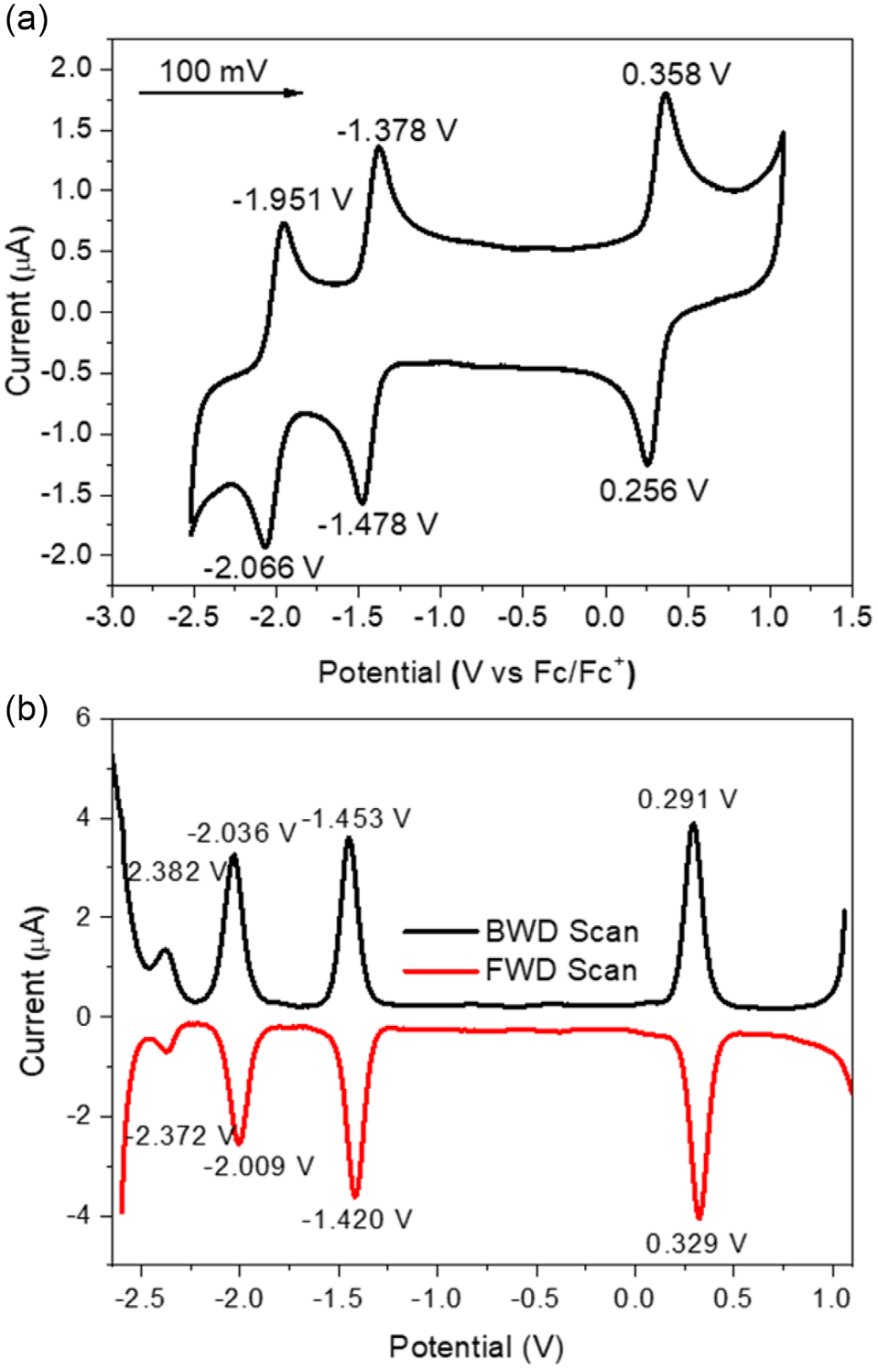
a) Cyclic and b) differential pulse voltammograms (vs Fc/Fc^+^) of 1.8 × 10^−3 ^M [*rac*‐**1**](PF_6_)_2_ recorded in 0.1 m [nBu_4_N]PF_6_/MeCN electrolyte using glassy carbon working electrode at 25 °C with a scanning rate of 100 mV s^−1^. For the CV the scan was initiated in the positive direction and both backward direction (BWD) scan and foward direction (FWD) scans were performed.

**Figure 3:**
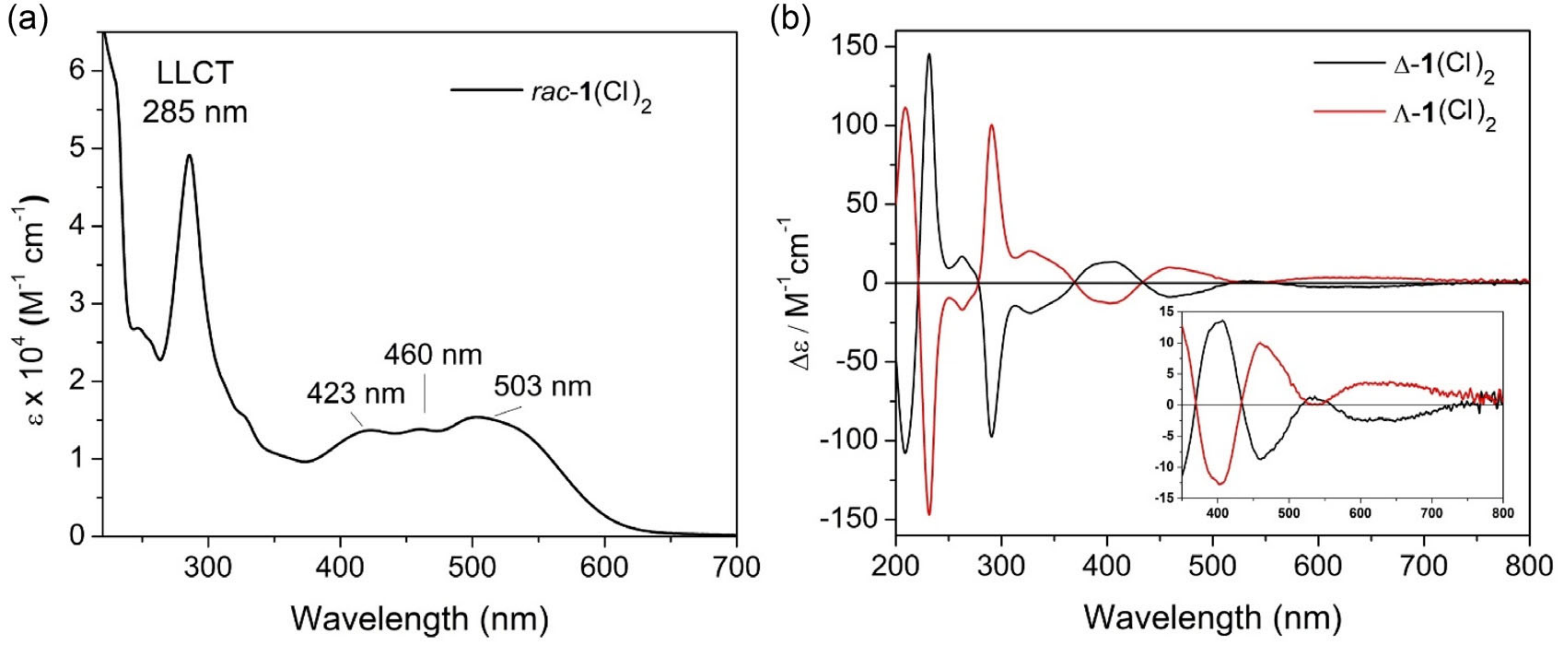
a) UV–visible spectra of [*rac*‐**1**](Cl)_2_ in a potassium phosphate buffer at pH 7.4 and b) the CD spectra of the [Δ‐**1**](Cl)_2_ and [Λ‐**1**](Cl)_2_ in water.

Cyclic voltammetry and differential pulse voltammetry (DPV) of [*rac*‐**1**](PF_6_)_2_ was measured using standard methodology involving an electrolytic solution composed of 0.1 m [nBu_4_N]PF_6_ dissolved in dry and degassed MeCN using different voltage scanning rate (**Figure** **2** and S5, Supporting Information). All values were referenced to F_c_/F_c_^+^ standard redox potential (−0.420 V).^[^ ^56^ ^]^ The [*rac*‐**1**](PF_6_)_2_ complex displays a full reversible redox couple in the oxidizing region at +0.307 V (Δ*E* 0.086 V, *I*_pc_/*I*_pa_ = 0.97), see **Figure** **3**. This is similar to what is observed for the related [Os(bpy)_3_]^2+^ and [Os(phen)_3_]^2+^ complexes, though **1** is slightly less resistant to oxidation than observed for [Os(dmbpy)_3_](PF_6_)_2_ (+0.372 V, Δ*E* 0.082 V) and considerable less than [Os(bpy)_3_](PF_6_)_2_ (+0.490 V, Δ*E* 0.076 V) measured under identical conditions.^[^ ^57^ ^]^ This suggests that the BIAN ligand in [*rac*‐**1**](PF_6_)_2_ is as strongly donating a ligand as dmbpy. DFT calculations on the gas phase B97D3 optimized model of [**1**]^2+^ reveal dominant Os d‐orbital character, with minor contributions from the BIAN ligand, whereas the B3LYP optimized model of [Os(bpy)_3_]^2+^ features only d‐orbital character.^[^ ^57^ ^]^ However, in contrast to the [Os(dmbpy)_3_](PF_6_)_2_ and [Os(phen)_3_](PF_6_)_2_ species, a set of fully reversible redox couples (−1.428 V, *I*_pc_/*I*_pa_ = 0.94 and −2.007 V, *I*_pc_/*I*_pa_ = 0.98) is observed at reducing potentials and is attributed to the ability of the BIAN ligand to act as a reversible electron reservoir. Several reports have suggested both one‐ and two‐electron reductions are supported by the acenaphthene framework, enabling efficient and stable radical delocalization.^[^ ^39^ ^]^ The DPV of [*rac*‐**1**](PF_6_)_2_ was able to resolve a fourth reversible redox couple (−2.375 V) not observed in the CV due to the reduction limit of the electrolyte (Figure 2b). In contrast, both [Os(dmbpy)_3_](PF_6_)_2_ and [Os(phen)_3_](PF_6_)_2_ show complicated irreversible reduction behavior past −1.6 V.^[^ ^57^ ^]^ The observation of third reduction peak for [*rac*‐**1**](PF_6_)_2_ suggests that the fourth redox couple is possibly dmbpy ligand based.

### 2.4 Photophysical Studies

The UV–vis absorption spectrum of [*rac‐* **1**](Cl)_2_ (recorded in an aqueous buffer at pH 7.4) shows bands characteristic of ligand‐centered and metal–ligand transitions, see Figure 3a. As a guide, time‐dependent DFT spectra were calculated from the geometry optimized models of the dicationic component of [Λ‐**1** and Λ‐**1**]^2+^ (Figure S6–7, Supporting Information). A good agreement (root mean derivation squared, RMDS 2.7) of the core metric parameters was obtained between the crystallographic solid state structure and DFT (Cam‐B3LYP with the solvent continuum model SMD), with the N_ax_–Os–N_ax_ bond angle showing the largest deviation (Table S8, Supporting Information). A comparison between the experimentally and time dependent density functional theory derived spectra reveals that the main bands are blueshifted in the latter, due to the neglect of SOC, which is observed in many types of Os complexes.^[^ ^58^ ^]^ The intense absorption band at 285 nm (48707 M^−1 ^cm^−1^) is assigned to a set of intra‐ and interligand–ligand charge transfers (LLCT) associated with the *π* to π* MOs associated with the BIAN ligand, and minor contributions from the *π* MOs of BIAN to the π* MOs of the dmbpy ligands (see Table S9 and Figure S6, S8, Supporting Information). The visible spectrum is dominated by three close lying or overlapping broad absorptions bands with maximum absorbance (*λ*_max_) at 423 nm (13518 M^−1 ^cm^−1^), 460 nm (13708 M^−1 ^cm^−1^), and 503 nm (15393 M^−1 ^cm^−1^), are attributed to a series of MLCT bands between the d‐orbitals of Os and π*‐MOs of the BIAN ligand and minor contributions from metal centered CTs. No detectable emission was observed when the solution was excited at wavelengths between 260 and 550 nm when [*rac*‐**1**](Cl)_2_ was dissolved in either an aqueous or organic solvent. The lack of emission in related Ru‐ and Ir‐*α*,*α*‐diimine systems has been attributed to either the presence of low lying dark states or arising due to lower energy emission in the NIR region.^[^ ^59^, ^60^ ^–^ ^61^ ^]^

The chiral resolution of the enantiopure forms of the complex was confirmed spectroscopically with near identical absorption spectra and CD spectra that are mirror images of each other (Figure 3b, Figure S3‐4, Table S1, Supporting Information). The CD spectrum recorded for each enantiomer is highly structured [Δ‐**1**](Cl)_2_ and [Λ‐**1**](Cl)_2_ and were assigned based on known affinity of the [Δ‐**1**](Cl)_2_ strong CD signals in the UV‐region of the enantiomers are related to the *π* → π* transitions of the dmbpy and BIAN ligands, while the lower intensity CD bands in the visible region correspond to the MLCT and LC transitions.^[^ ^62^ ^]^ The CD signals are induced by the helical assembly of the achiral ligands around the transition metal center.^[^ ^63^ ^]^ Bisignate signals are observed in the spectra attributed to excitation coupling of the electronic transition moments within the adjacent chromophores through‐space by Coulombic interactions.^[^ ^7^ ^,^ ^64^ ^,^ ^65^ ^]^ The transition bands at 434 nm for the Os(II) complex exhibit a near‐perfect Cotton effect, having through points close to the *λ*_max_, while the lower energy transition band is broad and weak and extends beyond 700 nm (Inset Figure 3a). The chromatographic method gave excellent resolution as seen in Figure S3–4, Supporting Information. The *Δε* values are presented in Table S1, Supporting Information.

### 2.5 Spectroscopic DNA Titrations

**Figure 4:**
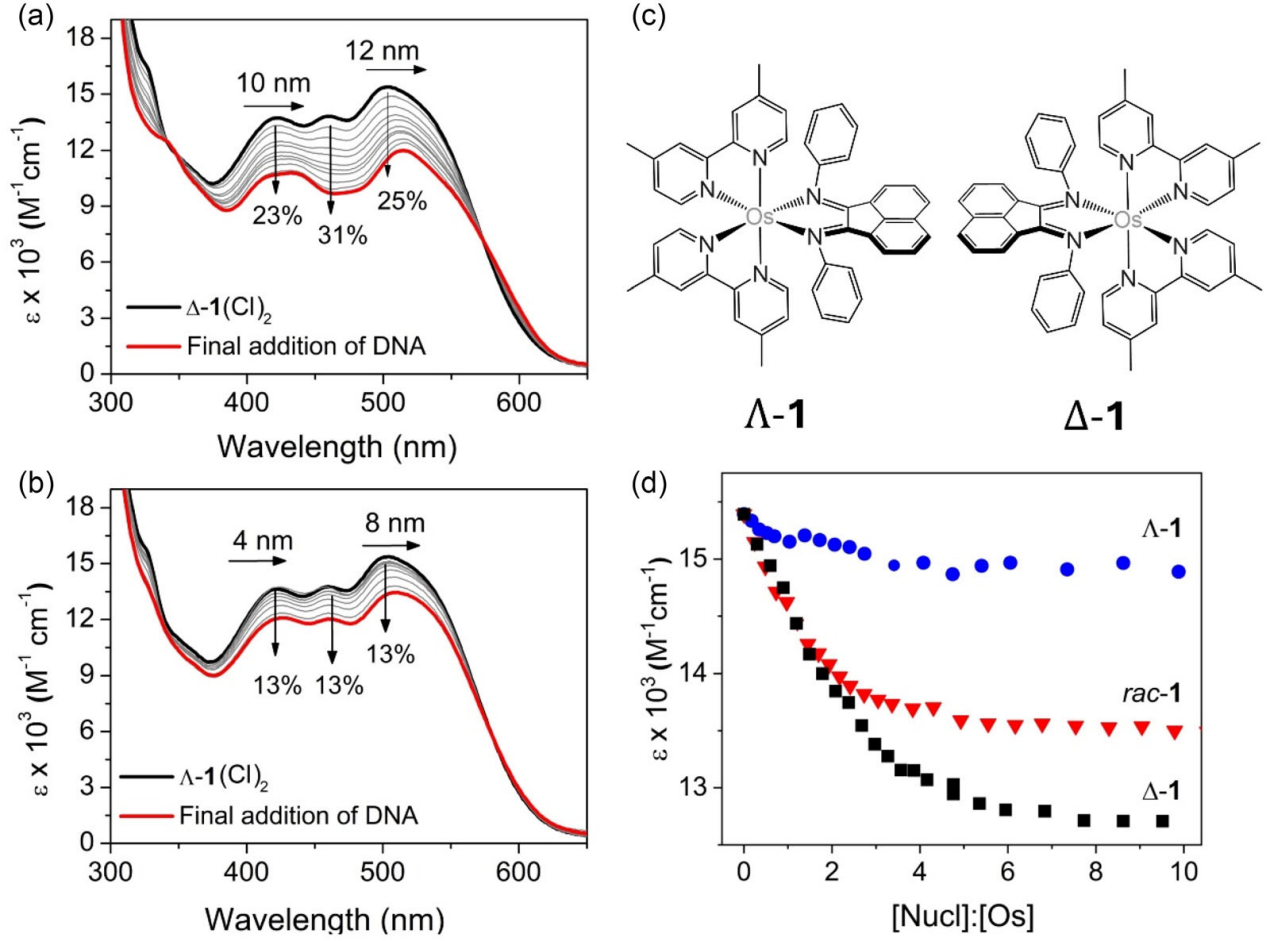
UV–vis DNA titration of a) [Δ‐**1**](Cl)_2_ and b) [Λ‐**1**](Cl)_2_ upon addition of increasing concentration of st‐DNA (0–126 μM) in 50 mM potassium phosphate buffered aqueous solution (pH 7.4). c) Structures of the enantiomers and d) change in absorbance at 503 nm upon addition of st‐DNA to [*rac*‐**1**](Cl)_2_, [Δ‐**1**](Cl)_2_, and [Λ‐**1**](Cl)_2_.

UV–vis absorbance titrations with double‐stranded salmon testes natural DNA (st‐DNA) were carried out with [*rac*‐**1**](Cl)_2_, [Δ‐**1**](Cl)_2_, and [Λ‐**1**](Cl)_2_ (**Figure** **4**a,b and Figure S9, Supporting Information). The delta enantiomer showed a significant change in absorbance with a decrease in intensity of 25% (503 nm) and 31% (460 nm), which were accompanied by a redshift in the absorbance maxima (*λ*_max_), see Figure 4a. A smaller hypochromism effect of 13% was observed upon the addition of st‐DNA to the [Λ‐**1**](Cl)_2_ species; again, these changes were accompanied by significant redshifts of 8 and 4 nm in the absorbance bands, respectively (Table S11, Supporting Information). For both enantiomers, the spectroscopic changes were accompanied by the appearance of isosbestic points at 580 nm (Δ‐**1**) and 575 nm (Λ‐**1**), see Figure 4a and b. The changes in the response to the addition of st‐DNA are highlighted in the difference spectra obtained by subtracting the spectrum recorded in the presence of DNA from that of the complex alone (see Figure S10, Supporting Information). Additionally, the titration of the racemic mixture showed similar changes to those observed for [Δ‐**1**](Cl)_2_, which suggests that the Δ‐enantiomer is largely responsible for the changes observed for the racemic mixture (Figure S9‐10, Supporting Information).

A summary of the titration results is given in **Table** **2**. The sensitivity of the binding to the enantiomer is highlighted in Figure 4d, which shows the change in absorbance measured as a function of added st‐DNA. The spectroscopic changes observed are similar to changes observed for other Ru‐ and Os‐polypyridyl complexes and suggest that in case of [*rac*‐**1**](Cl)_2_, it is the BIAN component that intercalates DNA.^[^ ^21^ ^,^ ^22^ ^,^ ^66^ ^,^ ^67^ ^]^ It is also notable that greater changes are observed for the delta enantiomer and suggests that the binding of the Λ‐**1** enantiomer may be inhibited by steric interactions.^[^ ^19^ ^]^ In the case of Pt(II) and Pd(II) supported BIAN complexes, DNA binding was determined indirectly using an ethidium bromide displacement assay^[^ ^44^ ^,^ ^45^ ^]^ and the changes in the UV–visible spectrum observed here for Δ‐**1** and Λ‐**1** are significantly more structured than those previously observed upon addition of st‐DNA to BIAN containing Mo(II) complexes.^[^ ^43^ ^]^ An attempt was made to determine the DNA binding constants *K*_b_ using the method reported by Bard,^[^ ^68^ ^]^ which revealed a binding constant on the order of 10^6 ^M^−1^ for Δ‐**1** (Table 2 and Figure S11, Supporting Information). This value is comparable with other metal‐based intercalators reported in the literature. However, it was not possible to determine a DNA binding constant for [Λ‐**1**](Cl)_2_, which is likely due to its significantly weaker interaction or the possible role of different binding modes.

**Table 2:** Results of the DNA titration with [*rac*‐**1**](Cl)_2_, [Δ‐**1**](Cl)_2_, and [Λ‐**1**](Cl)_2_.

@503 nm	*K*_*b*_ (10^5^ M^–1^)^[^ ^67^ ^]^	Binding site size (Base pair)	Plateau [Nu]:[Ru]
*rac* **‐1**	6.8 ± 1.0	1.0 ± 0.1	14
Λ‐**1**	–	–	11
Δ‐**1**	15.6 ± 2.7	1.6 ± 0.1	8

### 2.6 Circular and Linear Dichroism Spectroscopy

**Figure 5:**
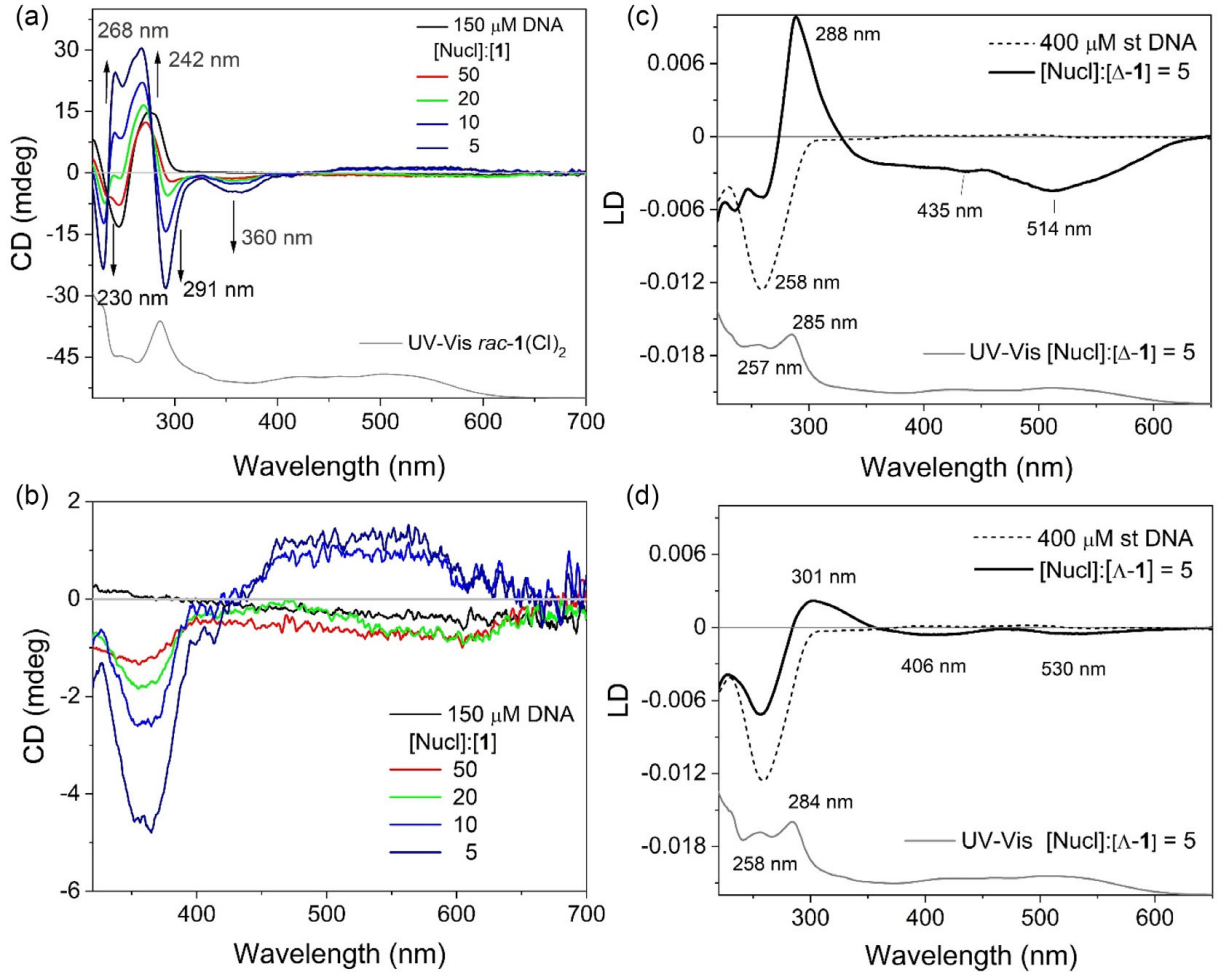
a,b) Circular dichroism spectra of [rac‐**1**](Cl)_2_ (30 μM) in the absence and presence of st‐DNA (150 μM, [Nucl]:[1] = 5). LD spectra of st‐DNA (400 μM) in the absence and presence of c) [Λ‐**1**](Cl)_2_ and d) [Δ‐**1**](Cl)_2_ at [Nucl]:[**1**]: of 5. All recorded in 50 mM potassium phosphate buffered aqueous solution.

CD measurements of [*rac*‐**1**](Cl)_2_ in the presence of different ratios of st‐DNA recorded in a 50 mM potassium phosphate buffer showed moderate changes in the spectrum, see **Figure** **5**a. The UV region showed the most significant changes, which is where the DNA absorbs indicating distortion in the DNA structure as well as induced chirality in the complex caused by interactions of the ancillary ligands near the deoxyribose units of the DNA helix, see Figure 5a. A stronger negative signal develops at 360 nm with increasing DNA concentration, and between 556 and 662 nm, and a weaker positive signal appears in the MLCT region, due to induced chirality. The broad weak signal extending beyond 650 nm is most pronounced at a [Nucl]:[**1**] of 5 and resembles the CD signal of the Λ‐**1** enantiomer. This reflects an enrichment of its presence in solution upon binding of the Δ‐**1** enantiomer (Figure 5b). This is a significant result as previous CD measurements of DNA binding of Pt(II) and Pd(II) BIAN complexes revealed subtle changes to the DNA region but no structurally induced changes to the complex.^[^ ^44^ ^,^ ^45^ ^]^

As CD cannot provide definitive information regarding the actual DNA binding mode, linear dichroism (LD) measurements in the presence of st‐DNA were also performed. These measurements educate the difference between the absorption of light polarized in a parallel and perpendicular (A_||_ ‐ A_⊥_) direction relative to the DNA macromolecules orientated in a couette cell. The LD spectrum of st‐DNA shows a characteristic negative band at 258 nm, which reflects the perpendicular orientation of the DNA nucleobases relative to the orientation of the aligned biomacromolecule in solution, see Figure 5c. In contrast, the LD spectrum recorded for [Δ‐**1**](Cl)_2_ in the presence of st‐DNA at a [Nucl]:[**1**] ratio of 5 is dominated by a strong positive band at 301 nm, which is characteristic of the location of the dmbpy ancillary ligands in the groove. Notably, there are also two strong negative bands at 435 nm and 513 nm, see Figure 5c, which complement the profile of the visible absorption bands in the presence of DNA. The appearance of these bands confirms intercalation of [Δ‐**1**](Cl)_2_, which places the chromophores parallel to the nucleobase orientation. The band positions indicate the orientation of the MLCT transition. The LD spectrum recorded for [Λ‐**1**](Cl)_2_ at the same ratio reveals weaker features, which absorb at different wavelengths. The observation of less intense bands mirrors the observation of the visible absorption titration and possibly indicates a greater role for the dmbpy ligands in this situation (Figure 5d). Notably, the bands in the visible/MLCT region of the spectrum are quite different. This may arise due to reduced intercalation and the location of the MLCT transition in the groove. In both cases, a decrease in the DNA absorbance is observed, which is attributed to overlap with the strongly absorbing ligand transitions in this region, though it may also be is the result of unwinding of the DNA helix.^[^ ^69^ ^]^ Overall, these measurements confirm that both enantiomers are capable of binding DNA through intercalation.

### 2.7 Thermal Denaturation Studies

**Figure 6:**
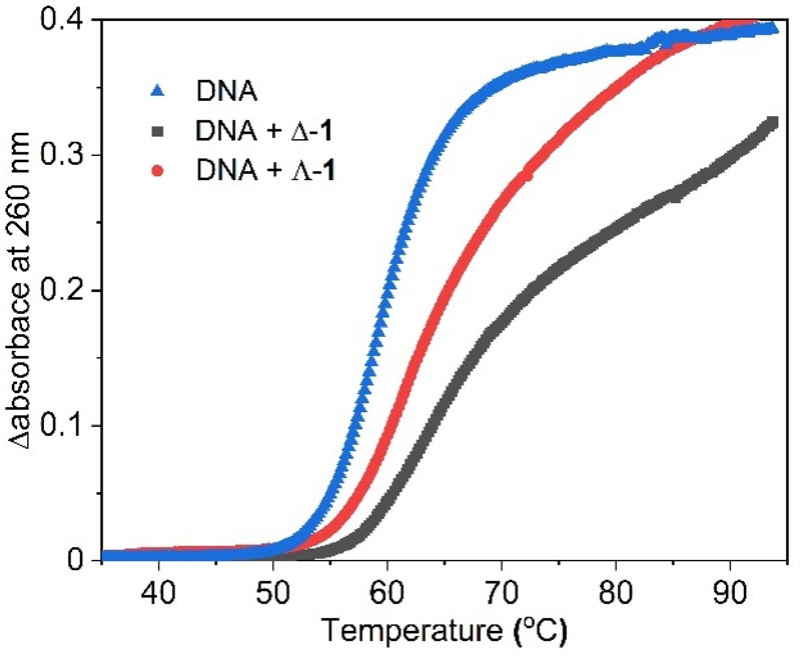
Thermal denaturation curves of st‐DNA (150 µM) in 1 mM aqueous sodium phosphate buffered solution with 2 mM NaCl at pH 7.4, in the absence and presence of [Λ‐**1**](Cl)_2_ and [Δ‐**1**](Cl)_2_ at a [Nucl]:[**1**] ratio of 20 after baseline correction.

Thermal denaturation studies were performed to investigate the impact of the bound [Λ‐**1**](Cl)_2_ and [Δ‐**1**](Cl)_2_ complexes on the DNA melting temperature (*T*_m_), defined as the temperature at which DNA is 50% denatured. The measurements were performed in the presence of the two enantiomers at [Nucl]:[**1**] ratio of 20 in a 1 mM potassium phosphate buffer with 2 mM NaCl at pH 7.4. The presence of the complex results in a significant change in the melting curve profile, which is also different for the two enantiomers, see **Figure** **6**. The change in the melting temperature from the derivative of the melting curve gave 2.1  ± 0.2 °C for the Λ‐**1** and 4.0 ± 0.6 °C for Δ‐**1** (Table S12, Supporting Information). This result is also in line with the observations in the spectroscopic titrations, where the greatest changes were observed for the Δ enantiomer and as such, it is expected that its binding would increase the melting temperature more significantly due to its increased binding affinity.

## 3 Conclusion

Employing standard synthetic procedures, a racemic dicationic osmium(II) bis–dipyridyl complex bearing a BIAN ligand has been prepared and chirally resolved into its enantiomers using chromatography. Cyclic voltammetry studies indicate fully reversible redox processes involving the Os center and BIAN ligand. UV–visible studies reveal a set of broad MLCT processes involving the metal and BIAN/dipyridyl ligands in which those associated with the BIAN component are highly sensitive to the solvent environment. The water‐soluble chloride complex enabled detailed studies of the interaction with st‐DNA employing visible absorption, CD, and LD assays and UV–vis denaturation studies. DNA spectroscopic titrations reveal a strong binding affinity of the delta enantiomer (*ca* 10^6 ^M^−1^) with significant redshifting of the absorption bands, while LD indicates intercalation primarily through the naphthalene component of the BIAN ligand with some minor interaction with the dipyridyl ligands. This order of magnitude of DNA binding is comparable to other Os(II) complexes, including [Os(TAP)_2_(dppz)]^2+^,^[^ ^21^ ^]^ and further highlights the potential of such BIAN‐containing complexes to expand the portfolio of DNA targeting agents. Future studies will explore the potential ROS activity of the complex and probe potential NIR excitations within a cellular environment.

## Supporting Information

The authors have cited additional references within the Supporting Information.^[^ ^70^, ^71^, ^72^, ^73^, ^74^, ^75^, ^76^, ^77^, ^78^, ^79^, ^80^, ^81^, ^82^ ^–^ ^83^ ^]^

## Conflict of Interest

The authors declare no conflict of interest.

## Supporting information

Supplementary Material

## Data Availability

The data that support the findings of this study are available from the corresponding author upon reasonable request.
