# Supplementary material for: DNA Binding Study of a Redox Active Enantiopure Bis(arylimino)acenaphthene (BIAN) Os(II) Bipyridine Complex
Source: Chembiochem. 2025 Oct 28;26(21):e202500536. doi: 10.1002/cbic.202500536 (PMC12596931; doi:10.1002/cbic.202500536)
Supplement: Supplementary file 1 — Supplementary Material [file CBIC-26-e202500536-s001.pdf]

Supporting Information

DNA Binding Study of a Redox Active Enantiopure Bis(arylimino)acenaphthene (BIAN) Os(II) Bipyridine Complex

Judit Fodor,[a] Susan J. Quinn[a]* and Andrew D. Phillips[a]*

Synthesis of [Os(4,4’-CH_3-2,2’bpy)$_2$BIAN](An)$_2$ An = PF_6 and Cl (1) 2
Experimental Methodology 2
Nuclear Magnetic Resonance (NMR) Spectroscopy 3
Elemental analysis 3
Mass Spectroscopy 3
Single Crystal X-ray Diffraction Studies 3
Cyclic and Differential Voltammetry 4
UV-Visible Absorption Spectroscopy 4
Circular Dichroism (CD) Spectroscopy 4
Linear Dichroism (LD) Spectroscopy 4
Preparation of the Buffer Solution 4
Preparation of Salmon Testes (st) DNA Solutions 4
UV-Visible Absorption DNA Titrations 5
Chiral Resolution Using Column Chromatography with CM and SP Sephadex® C-25 5
Computational Studies 6
NMR Spectroscopy 7
Enantiomeric Resolution 8
Solid-state structure 9
Cyclic Voltammetry 23
Computational Studies 24
UV-Vis Absorption DNA Titration 30
Circular Dichroism DNA Titrations 32
Thermal Denaturation Studies 33
References 33

WILEY - VCH

Synthesis of [Os(4,4'-CH_3-2,2'bpy)$_2$BIAN](An)$_2$ An = PF_6 and Cl (1)

Bis(4,4-CH_3-bpy)$_2$$OsCl_2$ (prepared according to a modified literature procedure [1]) 76 mg (0.12 mmol) and BIAN 40 mg (0.12 mmol) were suspended in ethylene glycol in a 20 mL MW vial and the reaction was heated to 180 °C for 2 hours. To the ethylene glycol water was added and the product was precipitated by adding ammonium hexafluorophosphate (NH_4PF_6) salt. The crude product (100 mg) was purified on a silica gel column, using an $CNCH_3$:H_2O: $NaNO_3$ (95:4:1) eluent. The dark red band was collected, and subsequently the solvent was removed. The afforded red material was dissolved in deionized water and precipitated out of solution using NH_4PF_6. In total 46 mg of the red product were recovered, giving a 33 % yield. For the enantiomeric resolution and the spectroscopic studies the water-soluble chloride complex was used. For this the PF_6^- salt of the (*rac*-**1**) complex was converted using Amberlite IRA-400 Cl ion exchange resin beads. The beads were soaked and washed with HPLC grade methanol prior to use. [*rac*-**1**](PF_6)$_2$ was dissolved in HPLC grade methanol or ethanol, then a spatula full (approximately 100 mg) of resin beads was added. Conversion was achieved in a couple of hours and [*rac*-**1**](Cl)$_2$ was collected by filtering the beads off and removing the solvent. The resulting solid was dried under dynamic vacuum overnight before being used in the DNA titrations. Stock solutions of [Λ/Δ-**1**]Cl_2 with approximately 3 mM concentration were prepared in deionised water.

^{1}H NMR (25 °C, 500 MHz, CD_3CN) δ 8.51 – 8.47 (s, 2H, H12), 8.33 – 8.24 (m, 6H, H3, H13, H9), 7.56 (ddd, *J* = 5.9, 2.0, 0.9 Hz, 2H, H10), 7.52 (dd, *J* = 8.3, 7.3 Hz, 2H, H2), 7.34 – 7.25 (m, 6H, H16, H7, H8), 7.18 - 7.14 (m, 4H, H16, H1), 7.12 (td, *J* = 7.8, 1.6 Hz, 2H, H6), 7.05 (ddd, *J* = 6.0, 1.9, 0.9 Hz, 2H, H5), 5.93 – 5.87 (m, 2H, H4), 2.76 (s, 6H, H11), 2.55 (s, 6H, H14).

^{13}C[2] NMR (126 MHz, CD_3CN) δ 176.25 (C=N), 159.39, 158.21, 153.55, 153.25, 151.84, 150.99, 147.55, 144.19, 132.81, 130.76, 130.64, 130.52, 130.38, 129.22, 129.03, 128.95, 128.91, 125.89, 125.31, 123.87, 121.69, 121.07, 21.31, 20.64

Elemental analysis: Found C, 54.17; H, 4.09; N, 7.98. $C_{48}H_40Cl_2N_6Os$ 1.15 H_2O. 1.4 NaCl requires C, 54.16; H,4.01; N 7.89%.

HRMS (MeCN/MeOH, *m/z*-ESI, positive mode): 442.1361 [M^{2+} Calc. 442.1414].

Experimental Methodology

All reagents and chemicals used in the synthetic procedures were purchased from Sigma Aldrich, Acros Organics (Fisher Scientific) and Fluorochem, and were used without further purification. HPLC grade and deuterated solvents for reactions and NMR were supplied from Sigma-Aldrich or Apollo Scientific. Millipore filtered deionised water was used in all purification steps and aqueous preparations. Chromatographic columns were performed using silica gel 60 (40-63 μm), or SP Sephadex or CM-25. The concentrations of metal complex, and DNA solutions were determined spectrophotometrically and made up in deionised water. The DNA titrations were carried out in potassium phosphate buffers prepared from the monobasic potassium phosphate (KH_2PO_4) and dibasic potassium phosphate (K_2HPO_4). The Salmon Testes (st-DNA) were purchased from Sigma Aldrich in their sodium salt forms.

WILEY-VCH

Nuclear Magnetic Resonance (NMR) Spectroscopy

All NMR spectra were recorded on a Varian VNMRS 500 MHz spectrometer. ^{1}H, $^{13}C\{^{1}H\}$, COSY, TOCSY, HMBC, and HSQC NMR spectra were measured using CD_3CN deuterated solvent. Chemical shifts for ^{1}H, $^{13}C\{^{1}H\}$ spectra were referenced to the relevant solvent peaks. All NMR data was processed and analysed using the MestReNova software suite from Mestrelab Research S. L.

Elemental analysis

Elemental analysis was performed on dried solids (2 to 3 mg) with an Exeter Analytical EA-1110 analyser.

Mass Spectroscopy

Electrospray ionization-mass spectra (ESI-MS) were obtained in positive ion mode on a Waters Alliance HT Micromass Quattro LCT instrument. The high-resolution mass spectra were obtained on a mass spectrometer with a TOF analyser operated by Dr Jimmy Muldoon. The accurate mass was reported within ±5 ppm.

Single Crystal X-ray Diffraction Studies

A suitable single crystal of [*rac*-***1***]$(PF_6)_2$ was removed from the growth vessel and solvent system and manipulated in a perfluoropolyalkylether oil matrix (F06206K, ABCR company) on a standard microscope slide. The crystals were mounted in the end of a glass fibre (diameter > 0.1 mm) attached to a copper pin fixed to a goniometer head which was placed in the Euler cradle, while maintaining a cold blanket of N_2 gas. Selected single crystals were kept under a 140 K gaseous flow of N_2 during the entire collection process. Diffraction data were collected on an Agilent SuperNova equipped with a 145mm Atlas-type CCD detector. The instrument utilised graphite monochromatic Mo-Kα (0.71073 Å) radiation. For data collection, optimised ω-scans were employed and data treatment, including cell indexing (using reflections measured from the entire data set), data reduction, gaussian-based adsorption corrections and space group determination was performed with the CrysAlias Pro application.[CrysAlisPRO, Oxford Diffraction /Agilent Technologies UK Ltd, Yarnton, England.] The structures were solved by a dual-space algorithm using the SHELXT program,[3] with the aid of successive difference Fourier maps and refined by full-matrix least-squares on F^2 for all data using SHELXL,[3] with the Olex2 suite of software.[4] Hydrogen atoms were added at calculated positions and refined using a riding model. Where electron density was sufficient, the hydrogen atoms were located manually, and the positions were allowed to be refined where the associated isotropic thermal displacement parameters were fixed to 1.2 times (1.5 times for methyl groups) to that of the connecting atom. Anisotropic thermal displacement parameters were used for all non-hydrogen atoms. Disorder within the molecules or solvates was treated using a split-occupancy model, where the occupancy factors were allowed to refine to non-integer values. In some instances, the bond distances of highly disordered components, in this case the PF_6 counterions were restrained to standard values, and some PF_6 ions had differing occupancies. Structural analysis was performed using the program PLATON[5] and figures were generated using Diamond v3.1. Collection and refinement data are given in table S1.

WILEY-VCH

Cyclic and Differential Voltammetry

The measurements were carried out using a gas-tight cell connected to a Gamry Instruments Interface 1000G potentiostat/galvanostat system. The three-electrode system consisted of a glassy carbon working electrode, a platinum wire as a counter electrode and an aqueous saturated calomel reference electrode or Ag/AgCl electrode as a reference. The supporting electrolyte was a 0.1M $[Bu_4N][PF_6]$ salt in degassed and dry acetonitrile. Solutions of the complexes (approximately 1 mM) were prepared in dry and degassed acetonitrile and degassed prior to measurements. Voltammograms were obtained with scan rates of 100, 200, 300, and 400 (mV/s) using a range of +1.8 V to -2.5 V, as shown in Figure S7. Differential pulse voltammetry was recorded in both directions (range 1.5 V to -2.2 V) with a step size of 10 mV and 1.0 s sample period with a pulse size of 25 mV and pulse time of 0.1 s.

UV-Visible Absorption Spectroscopy

UV-Vis spectra were recorded on a Varian 60 Agilent spectrometer equipped with a high intensity Xenon source in 0.2, 0.5 or 1 cm path length quartz cuvettes. Measurements were carried out at room temperature at a scan rate of 600 nm/min between 200 and 800 nm. Spectra of the “blank” solvents used were recorded and subtracted from the measurements. The molar extinction coefficients of the metal complexes were calculated using the Beer-Lambert Law.

Circular Dichroism (CD) Spectroscopy

CD spectra were recorded on a Jasco J-810 spectropolarimeter in a quartz cuvette with optical path lengths of 0.2-1 cm. CD measurements were taken at a data pitch of 1 nm, scanning speed of 100 nm min^{-1}, a response of 1 s, bandwidth of 1 nm, and data accumulation of 4-8 cycles.

Linear Dichroism (LD) Spectroscopy

LD spectra were recorded on a JASCO J-815 CD Spectropolarimeter fitted with a LD Accessory either in MilliQ water or a 50 mM potassium phosphate buffer. Spectra for the LD DNA titrations were accumulated over four cycles. LD measurements were taken at a data pitch of 1 nm, scanning speed of 100 nm min^{-1}, a response of 1 s, bandwidth of 1 nm, and data accumulation of four cycles.

Preparation of the Buffer Solution

Potassium phosphate buffers of 10- and 50-mM (pH 7.4) concentrations were prepared in deionised water using a combination of monobasic potassium phosphate (KH_2PO_4) and dibasic potassium phosphate (K_2HPO_4).

Preparation of Salmon Testes (st) DNA Solutions

Solutions of st-DNA were made up by dissolving 1-2 mg/mL of the sodium salt of the DNA in deionised water. The aqueous DNA solutions were sonicated between 15 to 30 minutes to assist the solvation process. The concentration of the solutions was determined by measuring the absorption at 260 nm, where DNA absorbs at the maximum. The concentration of the stock solution was calculated by using the Beer-Lambert equation ($A_{260}=\varepsilon cl$). The extinction coefficient at A_{260} (13200 M cm^{-1} per base pair or 6600 $M^{-1}cm^{-1}$ per base) was used. The purity of the DNA was checked by determining the ratio of absorption at 260nm and at 280nm with a value of circa 1.8 to 1.9, indicating purity. The lower values indicate that there are amino acid residues in the sample. A value greater than 1.9 can indicate partial DNA degradation and an increase in absorption originating from free base pairs.[6]

UV-Visible Absorption DNA Titrations

For the titrations, the complex stock solution was added to buffer (potassium phosphate buffers of 10- and 50-mM at pH 7.4) to make up a concentration in the 10-5 M range. The UV-Vis spectra were recorded between 200 and 800 nm. Aliquots of the ct-or st-DNA were added (approximately 10^{-6} M per addition) until no further change was observed in the intensity of the absorption exhibited by the metal complex. To ensure that binding saturation was reached, three independent measurements were carried out. The binding constants of the complexes were calculated using the *Bard*[7] method.

Chiral Resolution Using Column Chromatography with CM and SP Sephadex® C-25

Chromatographic resolution of the complexes was carried out using a Perspex column (1 m x 2 cm) containing CM or SP Sephadex C-25 as the stationary phase and a 0.1 M (-)-O, O'-dibenzoyl-L-tartrate acid monohydrate aqueous solution as the eluent. The chromatographic column was sealed by two adaptors (AC 26, GE Healthcare) and connected via capillary tubing to a to a Miniplus3 peristaltic pump (Gilson) to recycle the bands.

The Sephadex was soaked in MilliQ or the eluent overnight before filling the column and allowed to settle prior to loading. The flow rate was generally about 1-2 mL/min and reduced to 0.2 mL/min when the bands were recycled to avoid mixing in the capillary tube. After the Sephadex settled, the complex was loaded dissolved in water (10-30 mg), and allowed to adsorb at a continuous flow by addition of water or the tartrate solution. In some cases, adding Sephadex to the top of the loaded complex was necessary to avoid disturbing the adsorbed material. Introduction of the tartrate eluent onto the Sephadex equilibrated in water caused shrinkage and a reduction of the column length, therefore more Sephadex was added on top. However, equilibrating the Sephadex in the eluent gave better results and led to better adsorption of the complex as well, so this method was used in the second batch of the resolution procedures. Once the complex was adsorbed, the adapter was added on top, with the tubing placed in a reservoir containing the eluent. When the complex band reached the bottom, the tubing was connected to the pump and recycled. When streaking of the bands was observed, the system was disconnected after the bands were recycled, and fresh eluent was added until the bands reached the bottom of the column again. Streaking was greatly improved when the Sephadex was equilibrated in the eluent.

After multiple cycles, when separation was achieved, the bands were collected as fractions and were combined after the separation was confirmed the purity of the enantiomers.

The tartrate salt of the complex was converted by adding large amounts of the Na^+, K^+, or NH_4^+ PF_6^- salt to precipitate out the complex. The precipitate was collected either by filtration or by a centrifuge and washed with large amounts of water. This was followed by the conversion to the Cl^- salt using Amberlite IR-400 ion exchange beads. The complexes were either dissolved in methanol or ethanol and large amounts of the beads and a shaker were used to achieve the exchange. Generally, it took several days to reach full conversion. Removal of the excess tartrate, PF_6^-, and NaCl required thorough drying of the samples under vacuum followed by dissolving the complexes in a small amount of acetonitrile, stirring the solution, then cooling it in the fridge, and filtering it through celite. In some cases, it was necessary to repeat these steps.

WILEY-VCH

Computational Studies

The full structure of the dication of [Δ-**1**](PF_6)$_2$ was geometry optimized to an energy minimum in the gas phase showing no imaginary frequencies. The molecule adopted C_2 symmetry with the energy being equivalent to C_1. Subsequently the structure was reoptimized to an energy minimum using the SMD solvent continuum model for both water and dichloromethane. As suggested by the literature,[8] the long range separated hybrid functional CAM-B3LYP[9] with combined GDBJ3 empirical dispersion developed by Grimme with Becke-Johnson damping[10] was employed along with the basis set Def2SVP for C and H, Def2SVPD for N[11] and the pseudo potential basis set LanL2TZ(f) was used for Os[12] The final optimized geometry is shown in Figure S10 with a RMS of 2.93, selected metric parameters are given in table S9. TD-DFT derived simulation of the UV-VIS spectra using a solvent continuum of both water and DCM were obtained, but using the Def2TVZP basis set for C, H and N[11] and recording only the first 100 excited states. Triplet excitations were checked but found not to be present. The first 30 strong singlet-based excitations are listed in Table S10. Spectra data was processed with AOMIX v6.94[13] using a pseudo-Voight model which is a convolution of Gaussian and Lorentzian functions. Molecular orbitals were plotted at an isovalue of 0.01 au using the iQmol v3.1 viewing program and molecular geometry drawn using CYLview v2.0.[14] Geometry optimisation and TD-DFT simulation was preformed using the Gaussian 16 C.01 suite of programs.[15]

WILEY-VCH

Characterization

NMR Spectroscopy

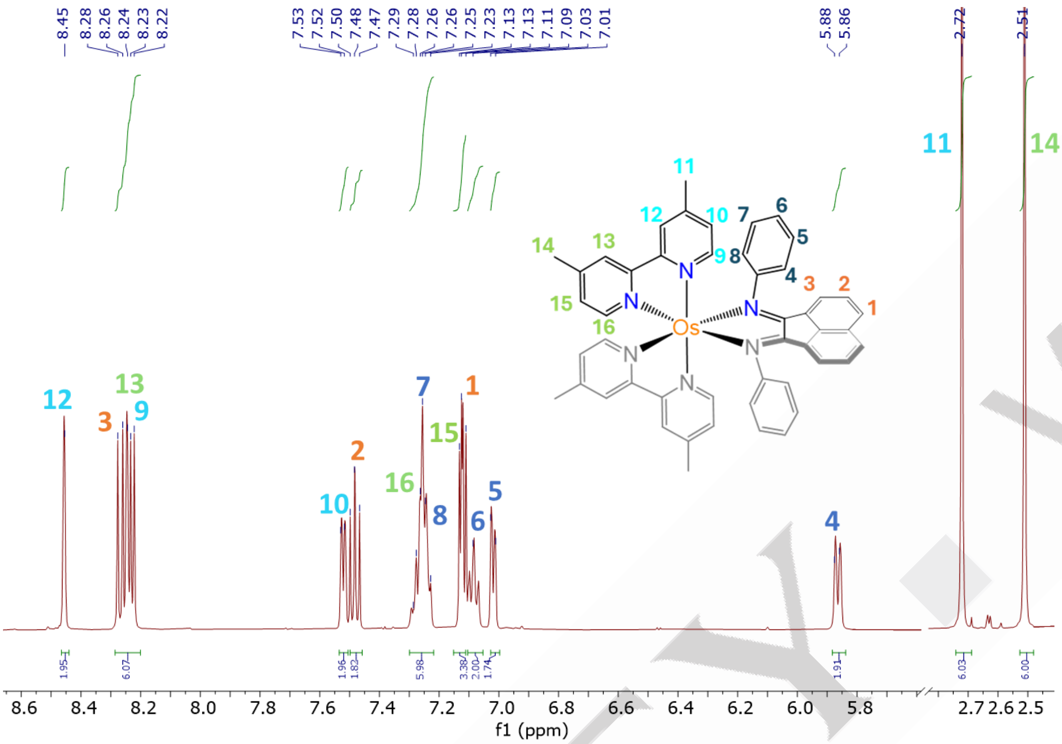

Figure S1. ^{1}H NMR (25 °C, 500 MHz, CD_3CN) spectrum of [*rac*-**1**](Cl)$_2$.

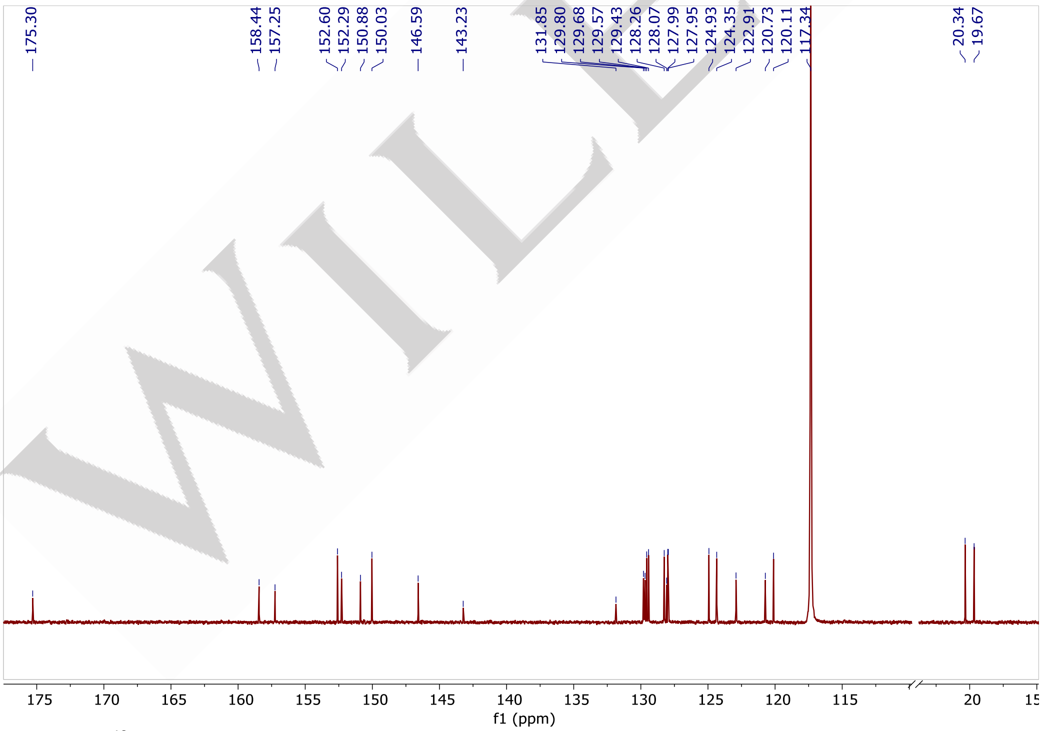

Figure S2. ^{13}C NMR (126 MHz, CD_3CN) spectrum of [*rac*-**1**](Cl)$_2$.

Enantiomeric Resolution

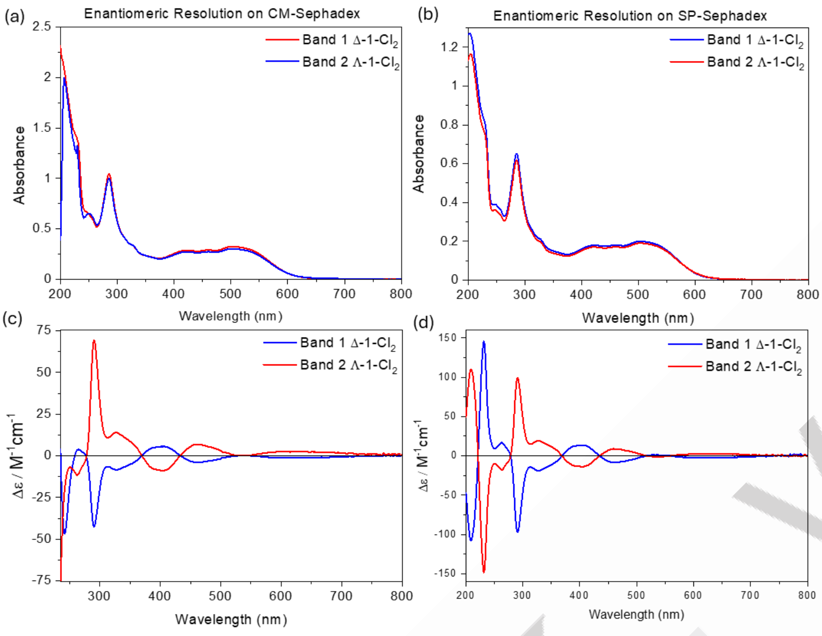

Figure S3. UV-Vis (a, b) and CD (c, d) spectra and of the resolved Δ-**1** and Λ-**1** enantiomers of [*rac*-**1**](Cl)$_2$ in water using SP-and CM-Sephadex.

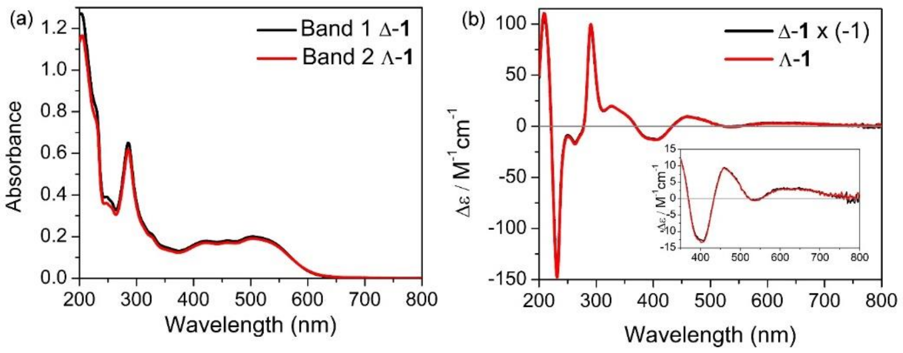

Figure S4. (a) UV-visible spectra of fractions obtained from chiral column. (b) Overlaid CD spectra of the Λ-**1** enantiomer and inversed Δ-**1** enantiomer of complexes [**1**](Cl)$_2$ in water. The inset show the spectra from 350 nm to 800 nm.

Table S1. Δε values for [Δ-**1**](Cl)$_2$ and [Λ-**1**](Cl)$_2$.

Os	209 nm	231 nm	263 nm	291 nm	327 nm	406 nm	460 nm	620 nm
Band 1 Δ	109	+146	+18	-99	-20	+14	-10	-4
Band 2 Λ	+113	-148	-17	+102	+21	-14	+11	+4

WILEY-VCH

Solid-state structure

Table S2. Crystal data and structure refinement for [*rac*-**1**]$(PF_6)_2$

Identification code	adp1179
Empirical formula	C_{50} H_{44} N_6 F_{12} P_2 Cl_4 Os
Molecular formula	$[C_{48}$ H_{40} N_6 $Os]^{2+}\{[F_6P]\}$
$_2$ 2($C1$ H_2 Cl_2)	
Formula weight	1350.85
Temperature	100(2) K
Wavelength	0.71073 Å
Crystal system	Monoclinic
Space group	C2/c (#15)
Unit cell dimensions	a = 38.5835(3) Å a= 90°.
	b = 12.02753(8) Å b= 104.8569(8)°.
	c = 23.5964(2) Å g = 90°.
Volume	10584.17(15) $Å^3$
Z	8
Density (calculated)	1.695 Mg/m^3
Absorption coefficient	2.757 mm^{-1}
F(000)	5344
Crystal size	0.270 × 0.210 × 0.120 mm^3
Theta range for data collection	2.691 to 33.071°.
Index ranges	$-55 \le h \le 57$, $-18 \le k \le 18$, $-35 \le l \le 35$
Reflections collected	170405
Independent reflections	18997 [R(int) = 0.0285]
Completeness to theta = 25.242°	99.8 %
Absorption correction	Gaussian
Max. and min. transmission	1.000 and 0.561
Refinement method	Full-matrix least-squares on F^2
Data / restraints / parameters	18997 / 0 / 737
Goodness-of-fit on F^2	1.059
Final R indices [I>2sigma(I)]	R_1 = 0.0266, wR_2 = 0.0641
R indices (all data)	R_1 = 0.0325, wR_2 = 0.0691
Extinction coefficient	n/a
Largest diff. peak and hole	2.563 and -1.617 e $Å^{-3}$

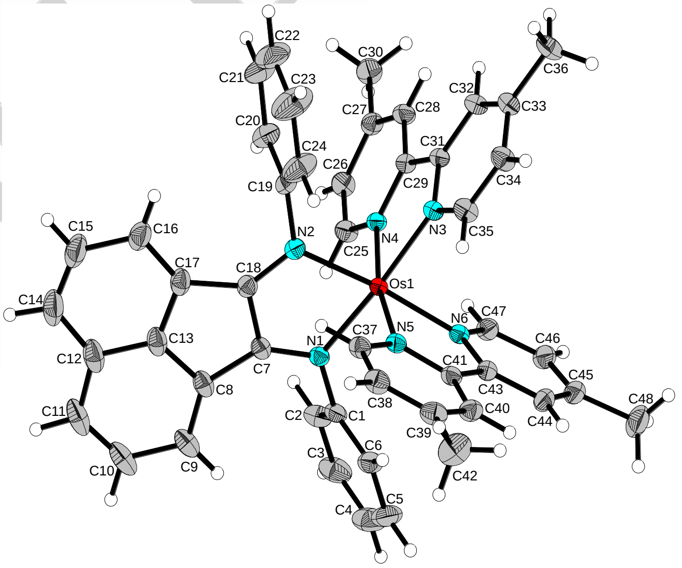

WILEY-VCH

Table S3. Atomic coordinates (x 10^4) and equivalent isotropic displacement parameters ($Å^2$x 10^3) for [*rac*-**1**]$(PF_6)_2$. U(eq) is defined as one third of the trace of the orthogonalized U^{ij} tensor.

	x	y	z	U(eq)
Os(1)	6361(1)	6553(1)	6296(1)	16(1)
C(1)	5764(1)	5053(2)	6549(1)	22(1)
C(2)	5807(1)	3948(2)	6721(1)	28(1)
C(3)	5642(1)	3560(2)	7142(1)	38(1)
C(4)	5435(1)	4263(3)	7379(1)	44(1)
C(5)	5393(1)	5367(3)	7201(1)	40(1)
C(6)	5558(1)	5773(2)	6786(1)	29(1)
N(1)	5942(1)	5468(1)	6126(1)	19(1)
C(7)	5831(1)	5126(2)	5581(1)	21(1)
C(8)	5548(1)	4396(2)	5253(1)	25(1)
C(9)	5265(1)	3824(2)	5370(1)	31(1)
C(10)	5055(1)	3143(2)	4918(1)	39(1)
C(11)	5123(1)	3037(2)	4382(1)	41(1)
C(12)	5409(1)	3637(2)	4242(1)	35(1)
C(13)	5613(1)	4314(2)	4690(1)	28(1)
C(14)	5516(1)	3621(2)	3712(1)	43(1)
C(15)	5801(1)	4246(3)	3649(1)	43(1)
C(16)	6009(1)	4920(2)	4106(1)	33(1)
C(17)	5913(1)	4945(2)	4629(1)	26(1)
C(18)	6052(1)	5477(2)	5198(1)	22(1)
N(2)	6320(1)	6136(1)	5440(1)	20(1)
C(19)	6583(1)	6412(2)	5129(1)	24(1)
C(20)	6835(1)	5614(2)	5083(1)	33(1)
C(21)	7100(1)	5891(3)	4801(1)	45(1)
C(22)	7107(1)	6939(3)	4570(2)	56(1)
C(23)	6853(1)	7723(3)	4614(2)	64(1)
C(24)	6590(1)	7461(2)	4902(1)	44(1)
N(3)	6801(1)	7596(1)	6415(1)	19(1)
C(25)	6762(1)	4341(2)	6562(1)	23(1)
C(26)	7055(1)	3644(2)	6671(1)	25(1)
C(27)	7398(1)	4078(2)	6736(1)	23(1)
C(30)	7723(1)	3346(2)	6849(1)	30(1)
C(28)	7426(1)	5227(2)	6693(1)	23(1)
C(29)	7122(1)	5892(2)	6585(1)	19(1)
C(31)	7131(1)	7106(2)	6546(1)	19(1)
C(32)	7444(1)	7728(2)	6645(1)	23(1)
C(33)	7430(1)	8881(2)	6624(1)	24(1)
C(36)	7762(1)	9580(2)	6739(1)	31(1)
C(34)	7092(1)	9367(2)	6503(1)	26(1)
C(35)	6788(1)	8724(2)	6399(1)	24(1)
N(4)	6788(1)	5454(1)	6515(1)	18(1)
N(5)	6005(1)	7848(1)	6168(1)	19(1)
C(37)	5824(1)	8258(2)	5642(1)	23(1)
C(38)	5613(1)	9200(2)	5584(1)	27(1)
C(39)	5580(1)	9770(2)	6078(1)	28(1)
C(42)	5362(1)	10814(2)	6033(1)	42(1)
C(40)	5760(1)	9341(2)	6624(1)	24(1)
C(41)	5967(1)	8391(2)	6659(1)	20(1)
C(43)	6162(1)	7874(2)	7212(1)	20(1)
C(44)	6161(1)	8270(2)	7765(1)	25(1)
C(45)	6354(1)	7725(2)	8265(1)	28(1)
C(48)	6370(1)	8171(3)	8866(1)	41(1)
C(46)	6533(1)	6752(2)	8188(1)	27(1)
C(47)	6523(1)	6389(2)	7629(1)	23(1)
N(6)	6349(1)	6945(1)	7143(1)	19(1)

P(1)	3468(1)	8588(1)	2496(1)	27(1)
F(1)	3082(1)	8483(1)	2642(1)	39(1)
F(2)	3849(1)	8714(2)	2356(1)	43(1)
F(3)	3663(1)	8318(2)	3162(1)	68(1)
F(4)	3467(1)	9871(1)	2645(1)	56(1)
F(5)	3266(1)	8830(2)	1832(1)	59(1)
F(6)	3463(1)	7296(2)	2359(1)	67(1)
P(2)	5000	981(1)	2500	26(1)
F(7) [a)]	4796(1)	-106(3)	2620(2)	50(1)
F(8) [a)]	5203(2)	2061(4)	2414(3)	63(1)
F(9) [a)]	5365(1)	316(6)	2663(3)	85(2)
F(10) [a)]	4943(2)	742(6)	1846(2)	75(3)
F(11) [a)]	4636(2)	1663(6)	2382(4)	95(3)
F(12) [a)]	5042(2)	1244(7)	3183(3)	93(3)
P(3)	7500	2500	5000	40(1)
F(13A) [b)]	7107(1)	2977(4)	4925(3)	83(2)
F(14A) [b)]	7536(2)	3176(4)	4439(2)	71(2)
F(15A) [b)]	7339(2)	1459(4)	4606(2)	65(1)
F(13B) [c)]	7268(2)	3129(5)	4434(2)	64(2)
F(14B) [c)]	7744(2)	1967(5)	4621(3)	61(2)
F(15B) [c)]	7243(2)	1462(6)	4847(4)	68(2)
C(49)	3447(1)	8385(2)	4418(1)	39(1)
Cl(1)	3529(1)	9632(1)	4812(1)	59(1)
Cl(2)	3811(1)	7479(1)	4621(1)	80(1)
C(50)	5602(1)	8362(3)	3552(1)	57(1)
Cl(3)	5784(1)	9329(1)	4100(1)	67(1)
Cl(4)	5399(1)	7262(1)	3828(1)	65(1)

[a)] s.o.f. set to 0.5 [b)] s.o.f. = 0.576(8) [c)] s.o.f. = 0.424(8) (s.o.f: **s**ite **o**ccupation **f**actor; the sum of the s.o.f.'s b) and c) is constrained to be 1)

Table S4. Bond lengths [Å] and angles [°] for [*rac*-**1**](PF_6)$_2$.

Os(1)-N(1)	2.0373(16)
Os(1)-N(2)	2.0472(16)
Os(1)-N(5)	2.0477(16)
Os(1)-N(6)	2.0661(16)
Os(1)-N(4)	2.0704(15)
Os(1)-N(3)	2.0705(16)
C(1)-C(6)	1.386(3)
C(1)-C(2)	1.387(3)
C(1)-N(1)	1.439(2)
C(2)-C(3)	1.391(3)
C(2)-H(2)	0.9500
C(3)-C(4)	1.375(4)
C(3)-H(3)	0.9500
C(4)-C(5)	1.390(4)
C(4)-H(4)	0.9500
C(5)-C(6)	1.385(3)
C(5)-H(5)	0.9500
C(6)-H(6)	0.9500
N(1)-C(7)	1.311(2)
C(7)-C(18)	1.455(3)
C(7)-C(8)	1.460(3)
C(8)-C(9)	1.376(3)
C(8)-C(13)	1.418(3)
C(9)-C(10)	1.423(3)
C(9)-H(9)	0.9500
C(10)-C(11)	1.362(4)
C(10)-H(10)	0.9500

WILEY-VCH

C(11)-C(12)	1.427(4)
C(11)-H(11)	0.9500
C(12)-C(13)	1.404(3)
C(12)-C(14)	1.413(4)
C(13)-C(17)	1.425(3)
C(14)-C(15)	1.372(5)
C(14)-H(14)	0.9500
C(15)-C(16)	1.421(3)
C(15)-H(15)	0.9500
C(16)-C(17)	1.378(3)
C(16)-H(16)	0.9500
C(17)-C(18)	1.459(3)
C(18)-N(2)	1.312(3)
N(2)-C(19)	1.436(3)
C(19)-C(24)	1.374(3)
C(19)-C(20)	1.391(3)
C(20)-C(21)	1.394(3)
C(20)-H(20)	0.9500
C(21)-C(22)	1.375(5)
C(21)-H(21)	0.9500
C(22)-C(23)	1.383(5)
C(22)-H(22)	0.9500
C(23)-C(24)	1.393(4)
C(23)-H(23)	0.9500
C(24)-H(24)	0.9500
N(3)-C(35)	1.359(2)
N(3)-C(31)	1.362(2)
C(25)-N(4)	1.349(2)
C(25)-C(26)	1.378(3)
C(25)-H(25)	0.9500
C(26)-C(27)	1.394(3)
C(26)-H(26)	0.9500
C(27)-C(28)	1.392(3)
C(27)-C(30)	1.499(3)
C(30)-H(30A)	0.9800
C(30)-H(30B)	0.9800
C(30)-H(30C)	0.9800
C(28)-C(29)	1.388(3)
C(28)-H(28)	0.9500
C(29)-N(4)	1.364(2)
C(29)-C(31)	1.464(3)
C(31)-C(32)	1.390(3)
C(32)-C(33)	1.389(3)
C(32)-H(32)	0.9500
C(33)-C(34)	1.390(3)
C(33)-C(36)	1.499(3)
C(36)-H(36A)	0.9800
C(36)-H(36B)	0.9800
C(36)-H(36C)	0.9800
C(34)-C(35)	1.374(3)
C(34)-H(34)	0.9500
C(35)-H(35)	0.9500
N(5)-C(37)	1.351(2)
N(5)-C(41)	1.371(2)
C(37)-C(38)	1.382(3)
C(37)-H(37)	0.9500
C(38)-C(39)	1.388(3)
C(38)-H(38)	0.9500
C(39)-C(40)	1.395(3)
C(39)-C(42)	1.500(3)

WILEY-VCH

C(42)-H(42A)	0.9800
C(42)-H(42B)	0.9800
C(42)-H(42C)	0.9800
C(40)-C(41)	1.386(3)
C(40)-H(40)	0.9500
C(41)-C(43)	1.468(3)
C(43)-N(6)	1.360(2)
C(43)-C(44)	1.389(3)
C(44)-C(45)	1.389(3)
C(44)-H(44)	0.9500
C(45)-C(46)	1.393(3)
C(45)-C(48)	1.501(3)
C(48)-H(48A)	0.9800
C(48)-H(48B)	0.9800
C(48)-H(48C)	0.9800
C(46)-C(47)	1.380(3)
C(46)-H(46)	0.9500
C(47)-N(6)	1.350(2)
C(47)-H(47)	0.9500
P(1)-F(4)	1.5826(17)
P(1)-F(6)	1.5860(19)
P(1)-F(5)	1.5877(18)
P(1)-F(3)	1.5918(19)
P(1)-F(2)	1.5943(16)
P(1)-F(1)	1.6179(16)
P(2)-F(10)	1.529(6)
P(2)-F(8)	1.556(4)
P(2)-F(9)	1.579(4)
P(2)-F(7)	1.586(3)
P(2)-F(11)	1.587(5)
P(2)-F(12)	1.608(7)
P(3)-F(15B)	1.578(6)
P(3)-F(15B)#1	1.578(6)
P(3)-F(15A)#1	1.588(4)
P(3)-F(15A)	1.588(4)
P(3)-F(13A)#1	1.589(4)
P(3)-F(13A)	1.589(4)
P(3)-F(14A)	1.590(4)
P(3)-F(14A)#1	1.590(4)
P(3)-F(14B)#1	1.592(4)
P(3)-F(14B)	1.592(4)
P(3)-F(13B)#1	1.595(5)
P(3)-F(13B)	1.595(5)
C(49)-Cl(2)	1.746(3)
C(49)-Cl(1)	1.750(3)
C(49)-H(49A)	0.9900
C(49)-H(49B)	0.9900
C(50)-Cl(3)	1.747(3)
C(50)-Cl(4)	1.747(3)
C(50)-H(50A)	0.9900
C(50)-H(50B)	0.9900
N(1)-Os(1)-N(2)	77.94(6)
N(1)-Os(1)-N(5)	89.38(6)
N(2)-Os(1)-N(5)	99.27(6)
N(1)-Os(1)-N(6)	97.01(6)
N(2)-Os(1)-N(6)	174.23(6)
N(5)-Os(1)-N(6)	77.74(6)
N(1)-Os(1)-N(4)	100.39(6)
N(2)-Os(1)-N(4)	86.90(6)

WILEY-VCH

N(5)-Os(1)-N(4)	169.40(6)
N(6)-Os(1)-N(4)	96.82(6)
N(1)-Os(1)-N(3)	175.41(6)
N(2)-Os(1)-N(3)	97.87(6)
N(5)-Os(1)-N(3)	93.17(6)
N(6)-Os(1)-N(3)	87.27(6)
N(4)-Os(1)-N(3)	77.37(6)
C(6)-C(1)-C(2)	121.22(19)
C(6)-C(1)-N(1)	119.27(18)
C(2)-C(1)-N(1)	119.49(18)
C(1)-C(2)-C(3)	119.2(2)
C(1)-C(2)-H(2)	120.4
C(3)-C(2)-H(2)	120.4
C(4)-C(3)-C(2)	120.2(2)
C(4)-C(3)-H(3)	119.9
C(2)-C(3)-H(3)	119.9
C(3)-C(4)-C(5)	120.0(2)
C(3)-C(4)-H(4)	120.0
C(5)-C(4)-H(4)	120.0
C(6)-C(5)-C(4)	120.7(2)
C(6)-C(5)-H(5)	119.6
C(4)-C(5)-H(5)	119.6
C(5)-C(6)-C(1)	118.7(2)
C(5)-C(6)-H(6)	120.7
C(1)-C(6)-H(6)	120.7
C(7)-N(1)-C(1)	118.45(16)
C(7)-N(1)-Os(1)	115.95(13)
C(1)-N(1)-Os(1)	125.60(12)
N(1)-C(7)-C(18)	114.90(17)
N(1)-C(7)-C(8)	136.06(19)
C(18)-C(7)-C(8)	108.88(17)
C(9)-C(8)-C(13)	119.5(2)
C(9)-C(8)-C(7)	136.0(2)
C(13)-C(8)-C(7)	104.51(18)
C(8)-C(9)-C(10)	117.8(2)
C(8)-C(9)-H(9)	121.1
C(10)-C(9)-H(9)	121.1
C(11)-C(10)-C(9)	122.8(2)
C(11)-C(10)-H(10)	118.6
C(9)-C(10)-H(10)	118.6
C(10)-C(11)-C(12)	120.9(2)
C(10)-C(11)-H(11)	119.6
C(12)-C(11)-H(11)	119.6
C(13)-C(12)-C(14)	116.2(3)
C(13)-C(12)-C(11)	115.8(2)
C(14)-C(12)-C(11)	128.0(2)
C(12)-C(13)-C(8)	123.2(2)
C(12)-C(13)-C(17)	123.1(2)
C(8)-C(13)-C(17)	113.69(18)
C(15)-C(14)-C(12)	120.8(2)
C(15)-C(14)-H(14)	119.6
C(12)-C(14)-H(14)	119.6
C(14)-C(15)-C(16)	122.9(2)
C(14)-C(15)-H(15)	118.6
C(16)-C(15)-H(15)	118.6
C(17)-C(16)-C(15)	117.6(3)
C(17)-C(16)-H(16)	121.2
C(15)-C(16)-H(16)	121.2
C(16)-C(17)-C(13)	119.4(2)
C(16)-C(17)-C(18)	135.7(2)

WILEY-VCH

C(13)-C(17)-C(18)	104.83(18)
N(2)-C(18)-C(7)	115.33(17)
N(2)-C(18)-C(17)	136.57(19)
C(7)-C(18)-C(17)	108.07(17)
C(18)-N(2)-C(19)	119.99(17)
C(18)-N(2)-Os(1)	115.49(13)
C(19)-N(2)-Os(1)	124.12(13)
C(24)-C(19)-C(20)	121.3(2)
C(24)-C(19)-N(2)	119.9(2)
C(20)-C(19)-N(2)	118.74(19)
C(19)-C(20)-C(21)	119.0(2)
C(19)-C(20)-H(20)	120.5
C(21)-C(20)-H(20)	120.5
C(22)-C(21)-C(20)	120.0(3)
C(22)-C(21)-H(21)	120.0
C(20)-C(21)-H(21)	120.0
C(21)-C(22)-C(23)	120.6(3)
C(21)-C(22)-H(22)	119.7
C(23)-C(22)-H(22)	119.7
C(22)-C(23)-C(24)	120.0(3)
C(22)-C(23)-H(23)	120.0
C(24)-C(23)-H(23)	120.0
C(19)-C(24)-C(23)	119.2(3)
C(19)-C(24)-H(24)	120.4
C(23)-C(24)-H(24)	120.4
C(35)-N(3)-C(31)	117.72(16)
C(35)-N(3)-Os(1)	125.25(13)
C(31)-N(3)-Os(1)	116.96(12)
N(4)-C(25)-C(26)	122.92(19)
N(4)-C(25)-H(25)	118.5
C(26)-C(25)-H(25)	118.5
C(25)-C(26)-C(27)	120.21(19)
C(25)-C(26)-H(26)	119.9
C(27)-C(26)-H(26)	119.9
C(28)-C(27)-C(26)	117.00(18)
C(28)-C(27)-C(30)	121.22(19)
C(26)-C(27)-C(30)	121.78(19)
C(27)-C(30)-H(30A)	109.5
C(27)-C(30)-H(30B)	109.5
H(30A)-C(30)-H(30B)	109.5
C(27)-C(30)-H(30C)	109.5
H(30A)-C(30)-H(30C)	109.5
H(30B)-C(30)-H(30C)	109.5
C(29)-C(28)-C(27)	120.48(18)
C(29)-C(28)-H(28)	119.8
C(27)-C(28)-H(28)	119.8
N(4)-C(29)-C(28)	121.86(17)
N(4)-C(29)-C(31)	114.37(16)
C(28)-C(29)-C(31)	123.76(17)
N(3)-C(31)-C(32)	121.76(17)
N(3)-C(31)-C(29)	114.30(16)
C(32)-C(31)-C(29)	123.92(17)
C(33)-C(32)-C(31)	120.43(18)
C(33)-C(32)-H(32)	119.8
C(31)-C(32)-H(32)	119.8
C(32)-C(33)-C(34)	117.02(18)
C(32)-C(33)-C(36)	121.96(19)
C(34)-C(33)-C(36)	121.02(19)
C(33)-C(36)-H(36A)	109.5
C(33)-C(36)-H(36B)	109.5

WILEY-VCH

H(36A)-C(36)-H(36B)	109.5
C(33)-C(36)-H(36C)	109.5
H(36A)-C(36)-H(36C)	109.5
H(36B)-C(36)-H(36C)	109.5
C(35)-C(34)-C(33)	120.89(19)
C(35)-C(34)-H(34)	119.6
C(33)-C(34)-H(34)	119.6
N(3)-C(35)-C(34)	122.16(19)
N(3)-C(35)-H(35)	118.9
C(34)-C(35)-H(35)	118.9
C(25)-N(4)-C(29)	117.52(16)
C(25)-N(4)-Os(1)	125.61(13)
C(29)-N(4)-Os(1)	116.77(12)
C(37)-N(5)-C(41)	117.46(16)
C(37)-N(5)-Os(1)	125.61(14)
C(41)-N(5)-Os(1)	116.76(12)
N(5)-C(37)-C(38)	122.88(19)
N(5)-C(37)-H(37)	118.6
C(38)-C(37)-H(37)	118.6
C(37)-C(38)-C(39)	120.09(19)
C(37)-C(38)-H(38)	120.0
C(39)-C(38)-H(38)	120.0
C(38)-C(39)-C(40)	117.48(19)
C(38)-C(39)-C(42)	121.7(2)
C(40)-C(39)-C(42)	120.9(2)
C(39)-C(42)-H(42A)	109.5
C(39)-C(42)-H(42B)	109.5
H(42A)-C(42)-H(42B)	109.5
C(39)-C(42)-H(42C)	109.5
H(42A)-C(42)-H(42C)	109.5
H(42B)-C(42)-H(42C)	109.5
C(41)-C(40)-C(39)	120.27(19)
C(41)-C(40)-H(40)	119.9
C(39)-C(40)-H(40)	119.9
N(5)-C(41)-C(40)	121.80(18)
N(5)-C(41)-C(43)	114.14(16)
C(40)-C(41)-C(43)	124.06(18)
N(6)-C(43)-C(44)	121.64(18)
N(6)-C(43)-C(41)	114.00(16)
C(44)-C(43)-C(41)	124.36(18)
C(43)-C(44)-C(45)	120.34(19)
C(43)-C(44)-H(44)	119.8
C(45)-C(44)-H(44)	119.8
C(44)-C(45)-C(46)	117.42(19)
C(44)-C(45)-C(48)	121.1(2)
C(46)-C(45)-C(48)	121.5(2)
C(45)-C(48)-H(48A)	109.5
C(45)-C(48)-H(48B)	109.5
H(48A)-C(48)-H(48B)	109.5
C(45)-C(48)-H(48C)	109.5
H(48A)-C(48)-H(48C)	109.5
H(48B)-C(48)-H(48C)	109.5
C(47)-C(46)-C(45)	119.90(19)
C(47)-C(46)-H(46)	120.1
C(45)-C(46)-H(46)	120.1
N(6)-C(47)-C(46)	122.71(19)
N(6)-C(47)-H(47)	118.6
C(46)-C(47)-H(47)	118.6
C(47)-N(6)-C(43)	117.89(17)
C(47)-N(6)-Os(1)	125.37(13)

WILEY-VCH

C(43)-N(6)-Os(1)	116.65(12)
F(4)-P(1)-F(6)	178.46(13)
F(4)-P(1)-F(5)	90.66(13)
F(6)-P(1)-F(5)	90.04(14)
F(4)-P(1)-F(3)	90.41(13)
F(6)-P(1)-F(3)	88.87(14)
F(5)-P(1)-F(3)	178.45(14)
F(4)-P(1)-F(2)	90.52(9)
F(6)-P(1)-F(2)	90.83(10)
F(5)-P(1)-F(2)	91.30(10)
F(3)-P(1)-F(2)	89.81(11)
F(4)-P(1)-F(1)	88.48(9)
F(6)-P(1)-F(1)	90.16(10)
F(5)-P(1)-F(1)	88.86(10)
F(3)-P(1)-F(1)	90.05(11)
F(2)-P(1)-F(1)	178.99(10)
F(10)-P(2)-F(8)	88.4(3)
F(10)-P(2)-F(9)	92.2(4)
F(8)-P(2)-F(9)	90.4(3)
F(10)-P(2)-F(7)	94.3(3)
F(8)-P(2)-F(7)	177.3(3)
F(9)-P(2)-F(7)	89.5(3)
F(10)-P(2)-F(11)	91.6(5)
F(8)-P(2)-F(11)	89.8(4)
F(9)-P(2)-F(11)	176.1(4)
F(7)-P(2)-F(11)	90.1(3)
F(10)-P(2)-F(12)	177.6(6)
F(8)-P(2)-F(12)	92.3(4)
F(9)-P(2)-F(12)	90.0(4)
F(7)-P(2)-F(12)	85.0(3)
F(11)-P(2)-F(12)	86.2(5)
F(15B)-P(3)-F(15B)#1	180.0(3)
F(15A)#1-P(3)-F(15A)	180.0
F(15A)#1-P(3)-F(13A)#1	90.0(3)
F(15A)-P(3)-F(13A)#1	90.0(3)
F(15A)#1-P(3)-F(13A)	90.0(3)
F(15A)-P(3)-F(13A)	90.0(3)
F(13A)#1-P(3)-F(13A)	180.00(12)
F(15A)#1-P(3)-F(14A)	88.9(3)
F(15A)-P(3)-F(14A)	91.1(3)
F(13A)#1-P(3)-F(14A)	89.5(3)
F(13A)-P(3)-F(14A)	90.5(3)
F(15A)#1-P(3)-F(14A)#1	91.1(3)
F(15A)-P(3)-F(14A)#1	88.9(3)
F(13A)#1-P(3)-F(14A)#1	90.5(3)
F(13A)-P(3)-F(14A)#1	89.5(3)
F(14A)-P(3)-F(14A)#1	180.0
F(15B)-P(3)-F(14B)	88.9(3)
F(15B)#1-P(3)-F(14B)	91.1(3)
F(14B)#1-P(3)-F(14B)	180.0
F(15B)-P(3)-F(13B)	89.9(4)
F(15B)#1-P(3)-F(13B)	90.1(4)
F(14B)#1-P(3)-F(13B)	89.7(3)
F(14B)-P(3)-F(13B)	90.3(3)
F(13B)#1-P(3)-F(13B)	180.0(5)
Cl(2)-C(49)-Cl(1)	111.42(16)
Cl(2)-C(49)-H(49A)	109.3
Cl(1)-C(49)-H(49A)	109.3
Cl(2)-C(49)-H(49B)	109.3
Cl(1)-C(49)-H(49B)	109.3

WILEY-VCH

H(49A)-C(49)-H(49B)	108.0
Cl(3)-C(50)-Cl(4)	111.14(18)
Cl(3)-C(50)-H(50A)	109.4
Cl(4)-C(50)-H(50A)	109.4
Cl(3)-C(50)-H(50B)	109.4
Cl(4)-C(50)-H(50B)	109.4
H(50A)-C(50)-H(50B)	108.0

Symmetry transformations used to generate equivalent atoms:
#1 -x+3/2,-y+1/2,-z+1

Table S5. Anisotropic displacement parameters ($Å^2$ x 10^3) for [*rac*-1](PF_6)$_2$. The anisotropic displacement factor exponent takes the form: -2p^2[h^2 a*^{2}U^{11} + ... + 2 h k a* b* U^{12}].

	U^{11}	U^{22}	U^{33}	U^{23}	U^{13}	U^{12}
Os(1)	14(1)	18(1)	17(1)	-1(1)	4(1)	0(1)
C(1)	15(1)	28(1)	22(1)	2(1)	4(1)	-2(1)
C(2)	21(1)	32(1)	33(1)	8(1)	7(1)	0(1)
C(3)	28(1)	47(1)	38(1)	18(1)	7(1)	-4(1)
C(4)	31(1)	70(2)	36(1)	16(1)	15(1)	-4(1)
C(5)	28(1)	61(2)	37(1)	2(1)	18(1)	3(1)
C(6)	20(1)	37(1)	29(1)	1(1)	8(1)	1(1)
N(1)	16(1)	21(1)	19(1)	1(1)	4(1)	1(1)
C(7)	19(1)	22(1)	21(1)	0(1)	2(1)	-1(1)
C(8)	22(1)	24(1)	25(1)	-1(1)	-2(1)	0(1)
C(9)	23(1)	29(1)	34(1)	3(1)	-3(1)	-4(1)
C(10)	28(1)	31(1)	47(1)	4(1)	-10(1)	-7(1)
C(11)	36(1)	33(1)	41(1)	-5(1)	-14(1)	-4(1)
C(12)	32(1)	32(1)	31(1)	-7(1)	-10(1)	5(1)
C(13)	27(1)	27(1)	24(1)	-3(1)	-4(1)	3(1)
C(14)	43(1)	48(1)	30(1)	-16(1)	-7(1)	8(1)
C(15)	43(1)	58(2)	24(1)	-14(1)	0(1)	13(1)
C(16)	32(1)	44(1)	21(1)	-5(1)	3(1)	8(1)
C(17)	27(1)	29(1)	20(1)	-3(1)	0(1)	5(1)
C(18)	21(1)	23(1)	19(1)	0(1)	3(1)	2(1)
N(2)	20(1)	22(1)	19(1)	1(1)	6(1)	2(1)
C(19)	27(1)	28(1)	21(1)	-2(1)	10(1)	-1(1)
C(20)	32(1)	38(1)	32(1)	1(1)	14(1)	6(1)
C(21)	39(1)	60(2)	43(1)	-6(1)	24(1)	4(1)
C(22)	61(2)	65(2)	59(2)	-7(2)	46(2)	-15(2)
C(23)	95(3)	43(2)	77(2)	7(2)	65(2)	-9(2)
C(24)	63(2)	30(1)	51(2)	6(1)	38(1)	3(1)
N(3)	16(1)	20(1)	20(1)	1(1)	4(1)	-1(1)
C(25)	20(1)	21(1)	29(1)	1(1)	7(1)	0(1)
C(26)	25(1)	20(1)	30(1)	1(1)	6(1)	2(1)
C(27)	21(1)	24(1)	24(1)	-1(1)	5(1)	5(1)
C(30)	25(1)	28(1)	37(1)	-1(1)	5(1)	9(1)
C(28)	17(1)	25(1)	28(1)	-1(1)	6(1)	2(1)
C(29)	17(1)	21(1)	20(1)	-1(1)	5(1)	0(1)
C(31)	16(1)	20(1)	21(1)	-1(1)	6(1)	-1(1)
C(32)	16(1)	25(1)	27(1)	1(1)	5(1)	-1(1)
C(33)	20(1)	25(1)	27(1)	-1(1)	5(1)	-4(1)
C(36)	22(1)	28(1)	42(1)	0(1)	5(1)	-7(1)
C(34)	24(1)	20(1)	35(1)	1(1)	5(1)	-1(1)
C(35)	23(1)	16(1)	32(1)	4(1)	5(1)	1(1)
N(4)	16(1)	19(1)	20(1)	0(1)	6(1)	0(1)
N(5)	15(1)	21(1)	21(1)	0(1)	3(1)	2(1)
C(37)	19(1)	28(1)	21(1)	4(1)	3(1)	1(1)
C(38)	23(1)	29(1)	28(1)	6(1)	3(1)	3(1)

WILEY-VCH

C(39)	22(1)	25(1)	36(1)	6(1)	8(1)	4(1)
C(42)	47(2)	34(1)	46(1)	9(1)	13(1)	20(1)
C(40)	21(1)	24(1)	29(1)	0(1)	8(1)	3(1)
C(41)	15(1)	22(1)	23(1)	0(1)	4(1)	1(1)
C(43)	17(1)	23(1)	21(1)	-2(1)	4(1)	1(1)
C(44)	24(1)	29(1)	23(1)	-4(1)	7(1)	3(1)
C(45)	24(1)	39(1)	21(1)	-4(1)	6(1)	1(1)
C(48)	46(1)	53(2)	23(1)	-9(1)	8(1)	7(1)
C(46)	23(1)	37(1)	19(1)	1(1)	4(1)	3(1)
C(47)	20(1)	29(1)	21(1)	2(1)	4(1)	4(1)
N(6)	15(1)	23(1)	20(1)	0(1)	4(1)	0(1)
P(1)	24(1)	27(1)	29(1)	1(1)	4(1)	-1(1)
F(1)	32(1)	39(1)	49(1)	6(1)	16(1)	-3(1)
F(2)	27(1)	49(1)	55(1)	-3(1)	14(1)	1(1)
F(3)	52(1)	110(2)	35(1)	20(1)	1(1)	20(1)
F(4)	46(1)	32(1)	98(2)	-16(1)	34(1)	-9(1)
F(5)	38(1)	106(2)	31(1)	10(1)	7(1)	19(1)
F(6)	51(1)	33(1)	116(2)	-19(1)	22(1)	-2(1)
P(2)	23(1)	27(1)	29(1)	0	8(1)	0
F(7) [a)]	54(2)	38(2)	71(3)	-6(2)	36(2)	-20(2)
F(8) [a)]	70(3)	45(2)	89(4)	-3(2)	48(3)	-28(2)
F(9) [a)]	37(2)	127(5)	100(4)	84(4)	35(2)	36(3)
F(10) [a)]	79(4)	124(6)	26(2)	-25(3)	16(2)	-53(4)
F(11) [a)]	48(3)	118(6)	134(6)	89(5)	52(4)	47(3)
F(12) [a)]	101(6)	138(7)	53(3)	-46(4)	43(4)	-73(5)
P(3)	35(1)	47(1)	35(1)	-9(1)	6(1)	-1(1)
F(13A) [b)]	47(2)	65(3)	134(6)	-4(3)	21(3)	6(2)
F(14A) [b)]	93(4)	70(3)	49(2)	-2(2)	15(2)	-19(3)
F(15A) [b)]	79(4)	56(2)	53(3)	-18(2)	6(2)	-12(2)
F(13B) [c)]	75(5)	67(3)	37(2)	-3(2)	-8(2)	18(3)
F(14B) [c)]	51(3)	82(4)	56(3)	-15(3)	24(3)	14(3)
F(15B) [c)]	48(3)	65(3)	90(6)	-21(4)	15(3)	-13(2)
C(49)	42(1)	32(1)	40(1)	-3(1)	3(1)	7(1)
Cl(1)	105(1)	30(1)	33(1)	2(1)	2(1)	-4(1)
Cl(2)	100(1)	87(1)	46(1)	5(1)	2(1)	63(1)
C(50)	65(2)	70(2)	33(1)	4(1)	9(1)	-20(2)
Cl(3)	74(1)	73(1)	43(1)	1(1)	-1(1)	-23(1)
Cl(4)	83(1)	39(1)	84(1)	1(1)	45(1)	4(1)

[a)] s.o.f. set to 0.5 [b)] s.o.f. = 0.576(8) [c)] s.o.f. = 0.424(8) (s.o.f: **s**ite **o**ccupation **f**actor; the sum of the s.o.f.'s b) and c) is constrained to be 1)

Table S6. Hydrogen coordinates (x 10^4) and isotropic displacement parameters (Å^{2}x 10^{3}) for [*rac*-**1**](PF_6)$_2$.

	x	y	z	U(eq)
H(2)	5948	3462	6553	34
H(3)	5672	2807	7266	45
H(4)	5321	3994	7664	53
H(5)	5250	5849	7366	48
H(6)	5531	6529	6667	34
H(9)	5212	3882	5741	37
H(10)	4858	2745	4992	47
H(11)	4979	2556	4098	49
H(14)	5388	3172	3396	52
H(15)	5862	4227	3284	52
H(16)	6208	5337	4052	40
H(20)	6828	4891	5241	39
H(21)	7275	5357	4768	54

H(22)	7288	7125	4379	67
H(23)	6857	8440	4447	77
H(24)	6418	8002	4941	52
H(25)	6531	4024	6519	28
H(26)	7023	2865	6702	30
H(30A)	7759	3086	6475	46
H(30B)	7688	2705	7085	46
H(30C)	7934	3768	7061	46
H(28)	7655	5558	6739	28
H(32)	7670	7362	6728	28
H(36A)	7762	10015	6388	47
H(36B)	7974	9097	6837	47
H(36C)	7768	10084	7068	47
H(34)	7071	10154	6492	32
H(35)	6561	9081	6312	29
H(37)	5844	7883	5297	27
H(38)	5490	9457	5205	33
H(42A)	5111	10622	6005	63
H(42B)	5376	11229	5682	63
H(42C)	5455	11274	6382	63
H(40)	5740	9702	6972	29
H(44)	6028	8919	7800	30
H(48A)	6129	8390	8886	62
H(48B)	6529	8820	8943	62
H(48C)	6463	7595	9159	62
H(46)	6661	6340	8519	32
H(47)	6644	5719	7585	28
H(49A)	3231	8024	4489	47
H(49B)	3398	8547	3993	47
H(50A)	5795	8069	3385	68
H(50B)	5423	8734	3232	68

Table S7. Torsion angles [°] for [*rac*-**1**]$(PF_6)_2$.

C(6)-C(1)-C(2)-C(3)	-0.4(3)
N(1)-C(1)-C(2)-C(3)	178.2(2)
C(1)-C(2)-C(3)-C(4)	0.7(4)
C(2)-C(3)-C(4)-C(5)	-0.5(4)
C(3)-C(4)-C(5)-C(6)	-0.1(4)
C(4)-C(5)-C(6)-C(1)	0.4(4)
C(2)-C(1)-C(6)-C(5)	-0.2(3)
N(1)-C(1)-C(6)-C(5)	-178.8(2)
C(6)-C(1)-N(1)-C(7)	-111.5(2)
C(2)-C(1)-N(1)-C(7)	69.8(2)
C(6)-C(1)-N(1)-Os(1)	68.8(2)
C(2)-C(1)-N(1)-Os(1)	-109.83(19)
C(1)-N(1)-C(7)-C(18)	-172.93(16)
Os(1)-N(1)-C(7)-C(18)	6.8(2)
C(1)-N(1)-C(7)-C(8)	1.7(3)
Os(1)-N(1)-C(7)-C(8)	-178.57(19)
N(1)-C(7)-C(8)-C(9)	5.4(4)
C(18)-C(7)-C(8)-C(9)	-179.7(2)
N(1)-C(7)-C(8)-C(13)	-173.5(2)
C(18)-C(7)-C(8)-C(13)	1.3(2)
C(13)-C(8)-C(9)-C(10)	1.7(3)
C(7)-C(8)-C(9)-C(10)	-177.1(2)
C(8)-C(9)-C(10)-C(11)	0.2(4)
C(9)-C(10)-C(11)-C(12)	-1.6(4)

WILEY-VCH

C(10)-C(11)-C(12)-C(13)	1.0(4)
C(10)-C(11)-C(12)-C(14)	179.9(3)
C(14)-C(12)-C(13)-C(8)	-178.1(2)
C(11)-C(12)-C(13)-C(8)	1.0(3)
C(14)-C(12)-C(13)-C(17)	-0.8(3)
C(11)-C(12)-C(13)-C(17)	178.3(2)
C(9)-C(8)-C(13)-C(12)	-2.4(3)
C(7)-C(8)-C(13)-C(12)	176.7(2)
C(9)-C(8)-C(13)-C(17)	-179.9(2)
C(7)-C(8)-C(13)-C(17)	-0.7(2)
C(13)-C(12)-C(14)-C(15)	-0.5(4)
C(11)-C(12)-C(14)-C(15)	-179.4(3)
C(12)-C(14)-C(15)-C(16)	1.3(4)
C(14)-C(15)-C(16)-C(17)	-0.8(4)
C(15)-C(16)-C(17)-C(13)	-0.5(3)
C(15)-C(16)-C(17)-C(18)	178.0(2)
C(12)-C(13)-C(17)-C(16)	1.3(3)
C(8)-C(13)-C(17)-C(16)	178.8(2)
C(12)-C(13)-C(17)-C(18)	-177.6(2)
C(8)-C(13)-C(17)-C(18)	-0.1(2)
N(1)-C(7)-C(18)-N(2)	-3.5(3)
C(8)-C(7)-C(18)-N(2)	-179.62(17)
N(1)-C(7)-C(18)-C(17)	174.62(17)
C(8)-C(7)-C(18)-C(17)	-1.5(2)
C(16)-C(17)-C(18)-N(2)	-0.1(4)
C(13)-C(17)-C(18)-N(2)	178.5(2)
C(16)-C(17)-C(18)-C(7)	-177.7(2)
C(13)-C(17)-C(18)-C(7)	1.0(2)
C(7)-C(18)-N(2)-C(19)	171.56(17)
C(17)-C(18)-N(2)-C(19)	-5.9(3)
C(7)-C(18)-N(2)-Os(1)	-1.4(2)
C(17)-C(18)-N(2)-Os(1)	-178.9(2)
C(18)-N(2)-C(19)-C(24)	107.7(3)
Os(1)-N(2)-C(19)-C(24)	-79.9(3)
C(18)-N(2)-C(19)-C(20)	-74.6(3)
Os(1)-N(2)-C(19)-C(20)	97.7(2)
C(24)-C(19)-C(20)-C(21)	0.0(4)
N(2)-C(19)-C(20)-C(21)	-177.6(2)
C(19)-C(20)-C(21)-C(22)	-0.4(4)
C(20)-C(21)-C(22)-C(23)	-0.2(5)
C(21)-C(22)-C(23)-C(24)	1.1(6)
C(20)-C(19)-C(24)-C(23)	0.9(5)
N(2)-C(19)-C(24)-C(23)	178.5(3)
C(22)-C(23)-C(24)-C(19)	-1.4(6)
N(4)-C(25)-C(26)-C(27)	-0.2(3)
C(25)-C(26)-C(27)-C(28)	0.8(3)
C(25)-C(26)-C(27)-C(30)	-179.4(2)
C(26)-C(27)-C(28)-C(29)	-0.7(3)
C(30)-C(27)-C(28)-C(29)	179.63(19)
C(27)-C(28)-C(29)-N(4)	-0.2(3)
C(27)-C(28)-C(29)-C(31)	178.71(19)
C(35)-N(3)-C(31)-C(32)	-1.6(3)
Os(1)-N(3)-C(31)-C(32)	-178.80(15)
C(35)-N(3)-C(31)-C(29)	176.96(17)
Os(1)-N(3)-C(31)-C(29)	-0.3(2)
N(4)-C(29)-C(31)-N(3)	-3.3(2)
C(28)-C(29)-C(31)-N(3)	177.73(18)
N(4)-C(29)-C(31)-C(32)	175.21(18)
C(28)-C(29)-C(31)-C(32)	-3.8(3)
N(3)-C(31)-C(32)-C(33)	1.2(3)

WILEY-VCH

C(29)-C(31)-C(32)-C(33)	-177.19(18)
C(31)-C(32)-C(33)-C(34)	0.2(3)
C(31)-C(32)-C(33)-C(36)	179.0(2)
C(32)-C(33)-C(34)-C(35)	-1.1(3)
C(36)-C(33)-C(34)-C(35)	-179.9(2)
C(31)-N(3)-C(35)-C(34)	0.6(3)
Os(1)-N(3)-C(35)-C(34)	177.60(16)
C(33)-C(34)-C(35)-N(3)	0.7(3)
C(26)-C(25)-N(4)-C(29)	-0.7(3)
C(26)-C(25)-N(4)-Os(1)	175.49(16)
C(28)-C(29)-N(4)-C(25)	0.9(3)
C(31)-C(29)-N(4)-C(25)	-178.14(17)
C(28)-C(29)-N(4)-Os(1)	-175.64(15)
C(31)-C(29)-N(4)-Os(1)	5.3(2)
C(41)-N(5)-C(37)-C(38)	1.3(3)
Os(1)-N(5)-C(37)-C(38)	-173.75(16)
N(5)-C(37)-C(38)-C(39)	0.2(3)
C(37)-C(38)-C(39)-C(40)	-1.4(3)
C(37)-C(38)-C(39)-C(42)	178.2(2)
C(38)-C(39)-C(40)-C(41)	1.1(3)
C(42)-C(39)-C(40)-C(41)	-178.5(2)
C(37)-N(5)-C(41)-C(40)	-1.6(3)
Os(1)-N(5)-C(41)-C(40)	173.90(15)
C(37)-N(5)-C(41)-C(43)	178.10(17)
Os(1)-N(5)-C(41)-C(43)	-6.4(2)
C(39)-C(40)-C(41)-N(5)	0.4(3)
C(39)-C(40)-C(41)-C(43)	-179.28(19)
N(5)-C(41)-C(43)-N(6)	0.2(2)
C(40)-C(41)-C(43)-N(6)	179.91(18)
N(5)-C(41)-C(43)-C(44)	-179.42(19)
C(40)-C(41)-C(43)-C(44)	0.3(3)
N(6)-C(43)-C(44)-C(45)	0.4(3)
C(41)-C(43)-C(44)-C(45)	180.0(2)
C(43)-C(44)-C(45)-C(46)	-2.7(3)
C(43)-C(44)-C(45)-C(48)	177.1(2)
C(44)-C(45)-C(46)-C(47)	2.2(3)
C(48)-C(45)-C(46)-C(47)	-177.6(2)
C(45)-C(46)-C(47)-N(6)	0.7(3)
C(46)-C(47)-N(6)-C(43)	-3.0(3)
C(46)-C(47)-N(6)-Os(1)	173.51(16)
C(44)-C(43)-N(6)-C(47)	2.5(3)
C(41)-C(43)-N(6)-C(47)	-177.15(17)
C(44)-C(43)-N(6)-Os(1)	-174.37(15)
C(41)-C(43)-N(6)-Os(1)	6.0(2)

Symmetry transformations used to generate equivalent atoms:
#1 -x+3/2,-y+1/2,-z+1

WILEY-VCH

Cyclic Voltammetry

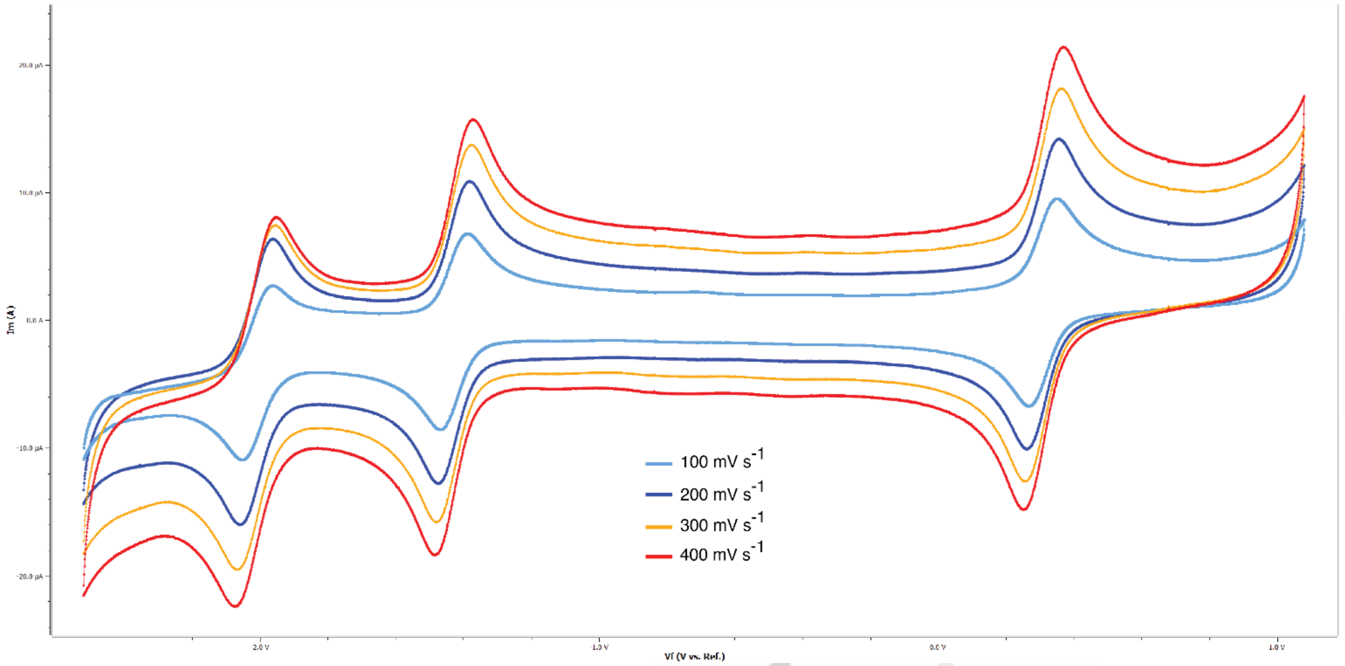

Figure S5. Cyclic voltammograms (vs Fc/Fc+) of 1.8 ×10^{-3} M [**rac-1**](PF_6)$_2$ recorded in 0.1 M [nBu_4N]PF_6/MeCN electrolyte using glassy carbon working electrode at 25 °C with a different scanning rates. For the CV the scan was initiated in the positive direction.

Computational Studies

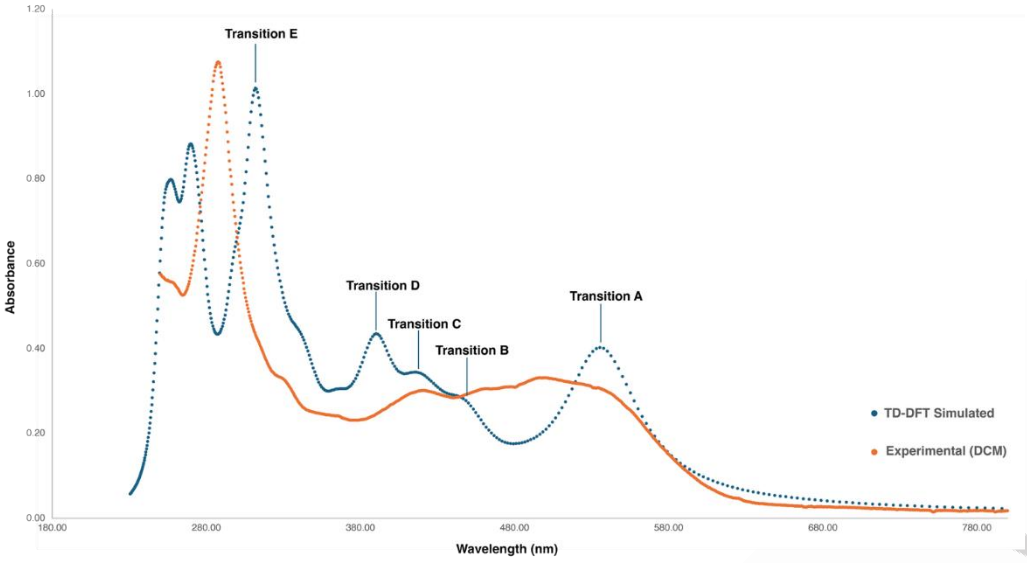

Figure S6. Comparison of experimental UV-Vis of **[rac-1](PF$_6$)$_2$** recorded in dry DCM versus TD-DFT simulated spectrum (SMD solvent continuum model with DCM) using the CAM-B3LYP level of theory. The TD-DFT spectrum has been shifted positively by 28 cm^{-1}.

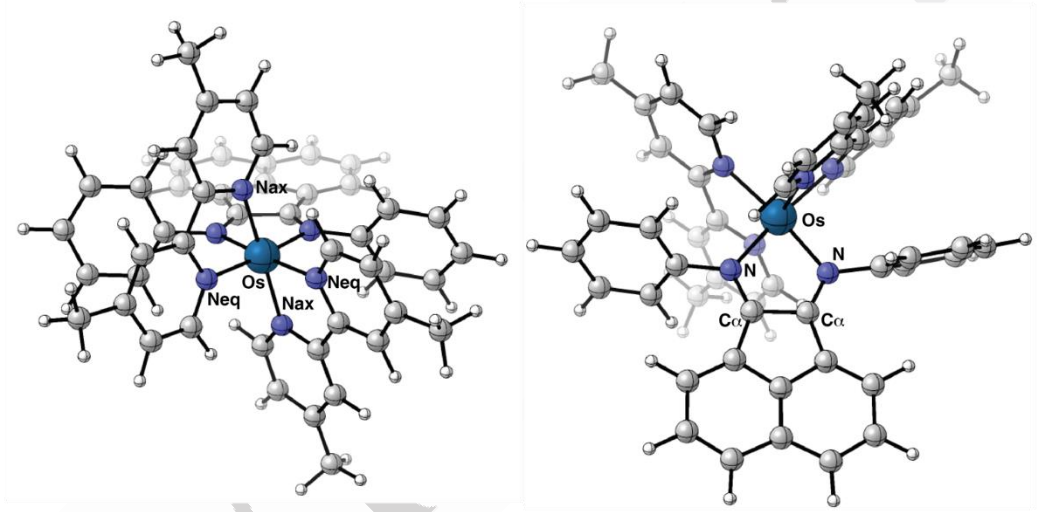

Figure S7. Finalised optimised geometry of the dication **[Δ-1]$^{2+}$** using DFT with CAM-B3LYP and SMD solvent continuum (DCM).

Table S8: Comparison of key metric parameters for DFT derived geometry optimized [Δ-1]$^{2+}$.

	Os-N (Å)	Os-N$_{eq}$ (Å)	Os-N$_{ax}$ (Å)	N-Os-N (°)	N$_{ax}$-Os-N$_{ax}$ (°)	N-C$_\alpha$ (°)	C$_\alpha$-C$_\alpha$ (°)
X-ray structure	2.047	2.070	2.066	77.94	169.40	1.312	1.455
Gas Phase	2.064	2.074	2.075	77.42	173.50	1.302	1.471
SMD(water)	2.047	2.073	2.072	77.62	172.32	1.303	1.458
SMD(DCM)	2.049	2.075	2.073	77.69	172.37	1.304	1.460

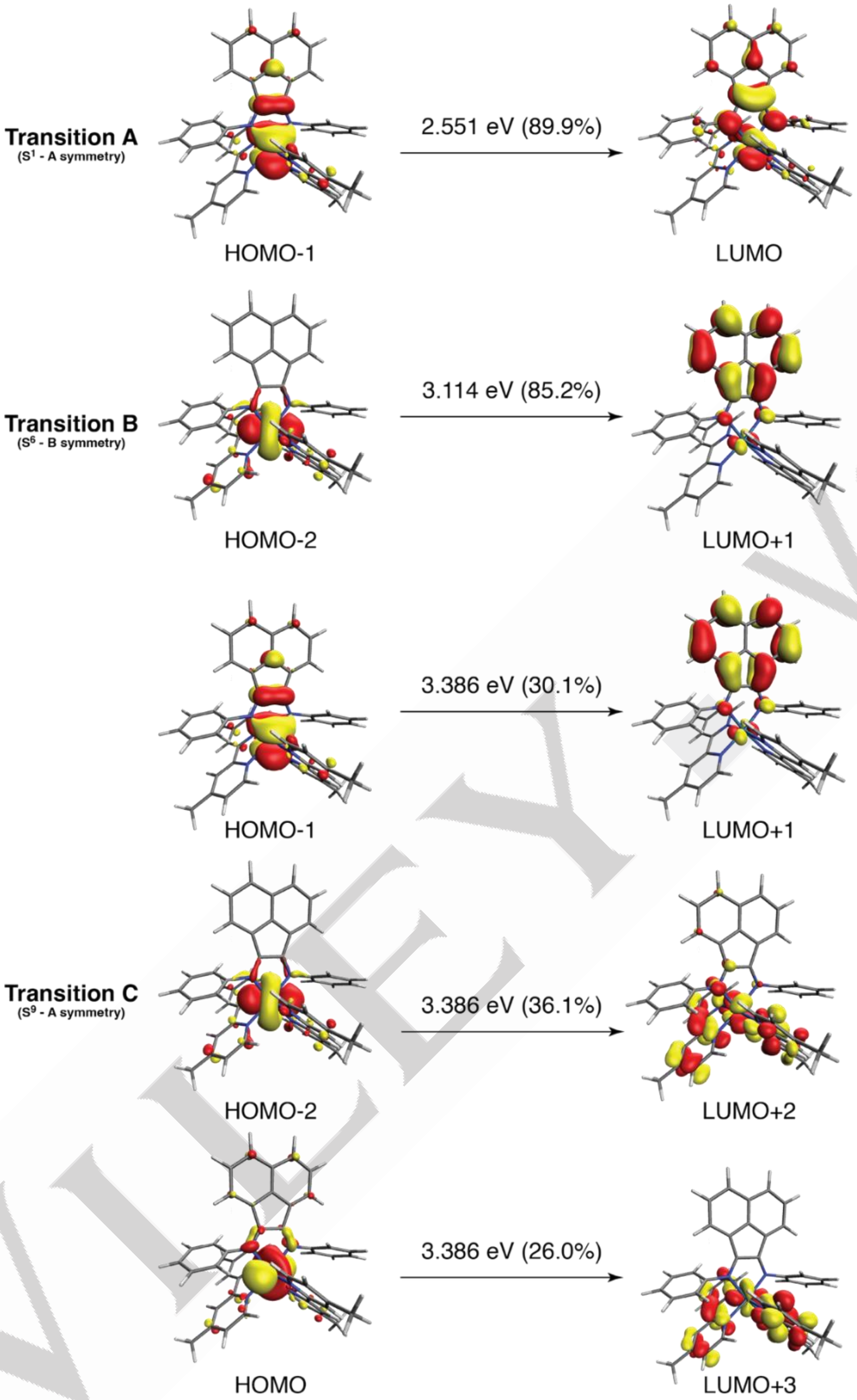

Figure S8. Molecular orbitals derived from the TD-DFT simulated singlet excitations (SMD solvent continuum model with DCM) using the CAM-B3LYP level of theory. MOs are drawn with an isovalue of 0.1 Å^{-3}.

WILEY-VCH

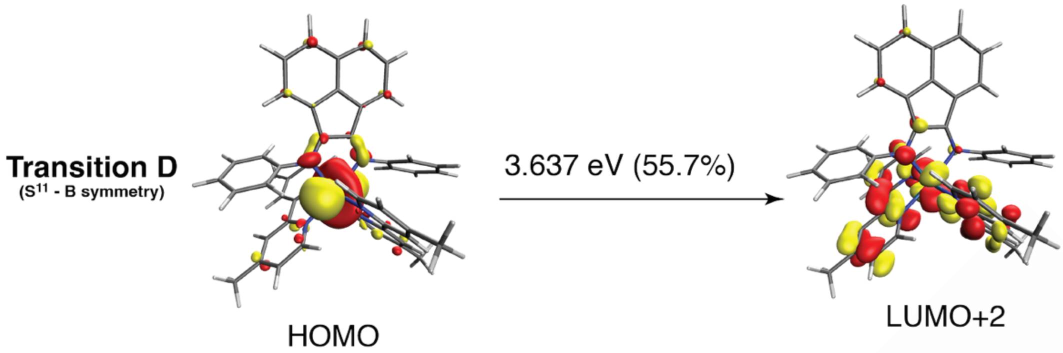

Figure S8 continued. Molecular orbitals derived from the TD-DFT simulated singlet excitations (SMD solvent continuum model with DCM) using the CAM-B3LYP level of theory. MOs are drawn with an isovalue of 0.1 Å^{-3}.

WILEY-VCH

Table S9: Complete listing of first 30 electronic transitions (f>0.01) from TD-DFT calculations with SMD solvation model (DCM) of [Δ-**1**]$^{2+}$.

Excited State	nm	eV	f	Assignment and % contribution	Symmetry
S^3	485.9	2.551	0.4291	HOMO-1→LUMO(89.9%)	A
S^4	444.5	2.789	0.0116	HOMO→LUMO+1(82.6%)<br>HOMO→LUMO(10.3%)	B
S^5	439.1	2.824	0.0151	HOMO→LUMO+2(84.3%)	A
S^6	398.1	3.114	0.1609	HOMO-2→LUMO+1(85.2%)	B
S^7	383.1	3.236	0.0098	HOMO-1→LUMO+1(48.2%)<br>HOMO-2→LUMO+2(30.4%)	A
S^8	376.1	3.297	0.0842	HOMO-1→LUMO+2(83.9%)	B
S^9	366.2	3.386	0.1397	HOMO-2→LUMO+2(36.1%)<br>HOMO-1→LUMO+1(31.9%)<br>HOMO→LUMO+3(26.0%)	A
S^{11}	340.9	3.637	0.3220	HOMO→LUMO+3(55.7%)<br>HOMO-1→LUMO+1(11.2%)<br>HOMO-2→LUMO+2 (11.0%)	B
S^{12}	331.4	3.741	0.0402	HOMO-3→LUMO(75.8%)<br>HOMO-1→LUMO+3(12.7%)	B
S^{13}	316	3.924	0.0277	HOMO→LUMO+4(88.6%)	B
S^{14}	315	3.936	0.0913	HOMO-2→LUMO+3(85.7%)	A
S^{15}	298.7	4.151	0.0272	HOMO-4→LUMO(75.0%)	A
S^{16}	295.2	4.2	0.0848	HOMO-2→LUMO+4(84.0%)	B
S^{18}	290.2	4.272	0.1171	HOMO-6→LUMO(79.2%)	B
S^{20}	286.2	4.332	0.0536	HOMO→LUMO+6(82.4%)	B
S^{22}	278.5	4.452	0.0356	HOMO-1→LUMO+5(43.7%)<br>HOMO-2→LUMO+6(15.6%)	B
S^{23}	277.5	4.468	0.0131	HOMO-3→LUMO+3(30.2%)<br>HOMO-2→LUMO+5(29.8%)<br>HOMO-2→LUMO+7(15.0%)<br>HOMO-7→LUMO+4(10.8%)	A
S^{24}	277.1	4.475	0.0727	HOMO-5→LUMO+3(44.0%)<br>HOMO-3→LUMO+8(14.3%)	B
S^{25}	275.8	4.495	0.0147	HOMO-3→LUMO+3(40.5%)<br>HOMO-2→LUMO+5(14.8%)<br>HOMO-2→LUMO+7(12.0%)	A
S^{26}	272.8	4.544	0.0154	HOMO-5→LUMO(80.4%)	A
S^{27}	271.3	4.571	0.0371	HOMO→LUMO+11(47.0%)<br>HOMO→LUMO+22(13.3%)	B
S^{28}	270	4.591	0.0600	HOMO-2→LUMO+5(27.2%)<br>HOMO-7→LUMO(12.6%)	A
S^{29}	269.8	4.595	0.0381	HOMO-2→LUMO+6(48.4%)<br>HOMO-1→LUMO+5(36.8%)	B
S^{30}	265.2	4.676	0.173	HOMO-2→LUMO+7(22.8%)<br>HOMO-7→LUMO(19.6%)	A

WILEY-VCH

Table S10: List of atomic coordinates for gas phase optimization of the dication [Δ-**1**]$^{2+}$.

Os	-0.06253900	-0.24224800	0.78089200
N	-1.62349500	-0.30007500	2.14543000
C	-2.18621900	0.77951200	2.71361200
H	-1.73558600	1.74168400	2.47025200
C	-3.28058300	0.69698200	3.55182900
H	-3.69349300	1.61173500	3.98039600
C	-3.85085100	-0.54854200	3.84119200
C	-3.24689500	-1.66187000	3.26071400
H	-3.64802900	-2.65425300	3.46201800
C	-2.14187300	-1.51803400	2.42810300
C	-1.44766300	-2.64240200	1.77672800
C	-1.80932000	-3.97740900	1.92378200
H	-2.65132600	-4.24206700	2.56197100
C	-1.11378700	-4.98333100	1.25431900
C	-0.05233100	-4.57751300	0.43554300
H	0.52909800	-5.30932900	-0.12778500
C	0.26610200	-3.23715600	0.33007200
H	1.07899700	-2.89753400	-0.31164800
N	-0.40558700	-2.27829300	0.99169800
N	1.29466400	-0.01758800	2.33315800
C	1.76422000	-1.02111000	3.09310000
H	1.34383300	-2.00818900	2.90074700
C	2.73235300	-0.83526500	4.06016600
H	3.07434300	-1.69210000	4.64310300
C	3.26707400	0.43948500	4.28234800
C	2.75861300	1.47480600	3.50084900
H	3.13594000	2.48674500	3.64175400
C	1.77874300	1.22859100	2.54468300
C	1.19075200	2.26619900	1.67979200
C	1.53969300	3.61194700	1.72260100
H	2.28765200	3.95384100	2.43673100
C	0.95171400	4.52988900	0.85338900
C	0.01033800	4.02550200	-0.05276400
H	-0.48154600	4.68401800	-0.77064800
C	-0.30147900	2.67944900	-0.04818200
H	-1.02061300	2.26303400	-0.75344100
N	0.26434300	1.80717800	0.80465700
C	-0.59960900	-0.46866700	-2.04266100
C	0.86229800	-0.33397300	-1.94825400
N	-1.23574900	-0.41560200	-0.90802200
N	1.33461300	-0.25261700	-0.73782000
C	-2.12470100	-0.73079200	-4.17522700
C	-0.95623500	-0.59487900	-3.45730700
C	0.28228600	-0.52481600	-4.15584300
C	0.37965200	-0.60445400	-5.54940000
C	-0.84239900	-0.74700000	-6.26235500
C	-2.04560700	-0.80242400	-5.58882200
H	-3.09659200	-0.78737300	-3.68507900
C	1.41255000	-0.36622300	-3.30488500
C	1.68983100	-0.53399500	-6.09759900
H	-0.82513100	-0.81230100	-7.35233200
H	-2.97010800	-0.90950000	-6.15842000
C	2.78847200	-0.39306100	-5.27446500
C	2.67029500	-0.30333600	-3.86463400
H	1.82433500	-0.59283500	-7.17975400
H	3.78382100	-0.34395100	-5.71885000
H	3.56528200	-0.18386700	-3.25410900
C	-2.63995700	-0.66882700	-0.83895200
C	-3.51181800	0.32871500	-0.40536800
C	-3.11900900	-1.94731300	-1.12664300
C	-4.86786300	0.04837000	-0.27429600
H	-3.12758100	1.32249600	-0.17350400
C	-4.47598800	-2.22236000	-0.98479300
H	-2.42510500	-2.72279700	-1.45638500
C	-5.35238400	-1.22694000	-0.55936100

WILEY-VCH

H	-5.55262400	0.83361200	0.05103500
H	-4.85100700	-3.22203300	-1.21179000
H	-6.41681800	-1.44346300	-0.45447800
C	2.71772200	0.01795800	-0.50417200
C	3.51377400	-0.91761100	0.15478600
C	3.24157200	1.25988900	-0.86465500
C	4.84083800	-0.61341400	0.43946200
H	3.09384000	-1.88227300	0.44133600
C	4.56802800	1.56002000	-0.56866600
H	2.60570200	1.98782000	-1.37200500
C	5.36973300	0.62570300	0.08267300
H	5.46815700	-1.35165300	0.94226900
H	4.97836300	2.53088200	-0.85250600
H	6.41110400	0.86116900	0.30851300
C	-1.47267300	-6.42907100	1.40568900
H	-1.51673200	-6.92912900	0.42768600
H	-0.70710900	-6.95163900	2.00059800
H	-2.43862000	-6.55986000	1.90953700
C	-5.05137800	-0.66863500	4.72795100
H	-4.87589300	-0.17166600	5.69334300
H	-5.92097500	-0.17680100	4.26563900
H	-5.31450400	-1.71620600	4.92065700
C	4.33354100	0.66902100	5.30800900
H	4.01922900	0.28818800	6.29085900
H	5.25338000	0.13054100	5.03285700
H	4.57917400	1.73329600	5.41168700
C	1.29793800	5.98619000	0.88787400
H	1.47500300	6.37392100	-0.12540500
H	0.46401100	6.56615000	1.31372200
H	2.18931900	6.18004700	1.49771300

WILEY-VCH

UV-Vis Absorption DNA Titration

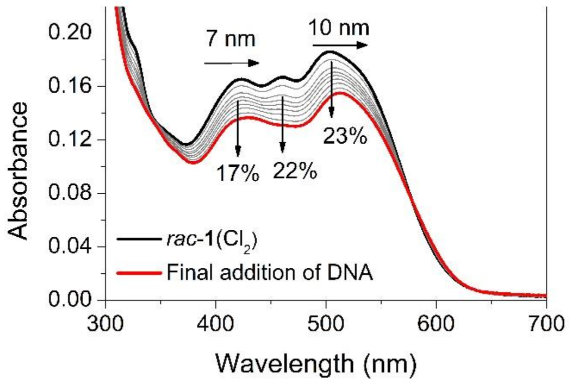

Figure S9. UV-Vis DNA titration of [*rac*-**1**]$(Cl)_2$ upon addition of increasing concentration of st-DNA (0-129 µM) in 50 mM potassium phosphate buffered aqueous solution (pH 7.4).

Table S11. Results of the UV-Vis DNA titrations for rac-, Λ- and [Δ-**1**]$(Cl)_2$.11

	Δ nm @ 503 nm	Δ nm @ 460 nm	Δ nm @ 423 nm	Δ abs (%) @ 503 nm	Δ abs (%) @ 460 nm	Δ abs (%) @ 423 nm
***Rac*-1**	10		7	13	16	13
**Λ-1**	8	4	4	3	3	2
**Δ-1**	12		10	15	20	12

WILEY-VCH

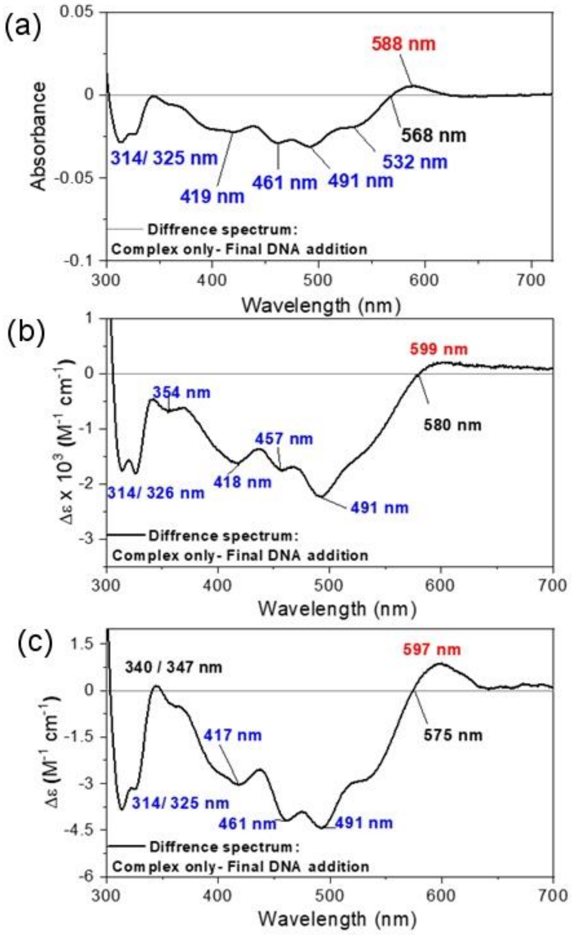

Figure S10. Difference spectra: Spectrum of the complex minus the spectrum after the final aliquot of st-DNA is added when the DNA binding reached completion, for (a) [*rac*-**1**](Cl)$_2$**,** (b) [Λ-**1**](Cl)$_2$ and (c) [Δ-**1**](Cl)$_2$.

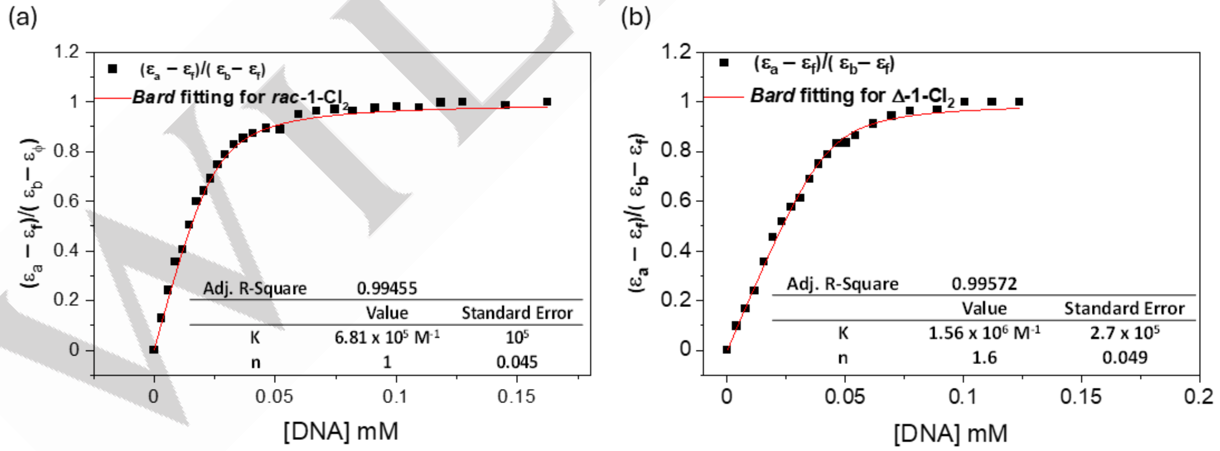

Figure S11. Bard fitting plots for (a) [*rac*-**1**](Cl)$_2$ and (b) [Δ-**1**](Cl)$_2$

Circular Dichroism DNA Titrations

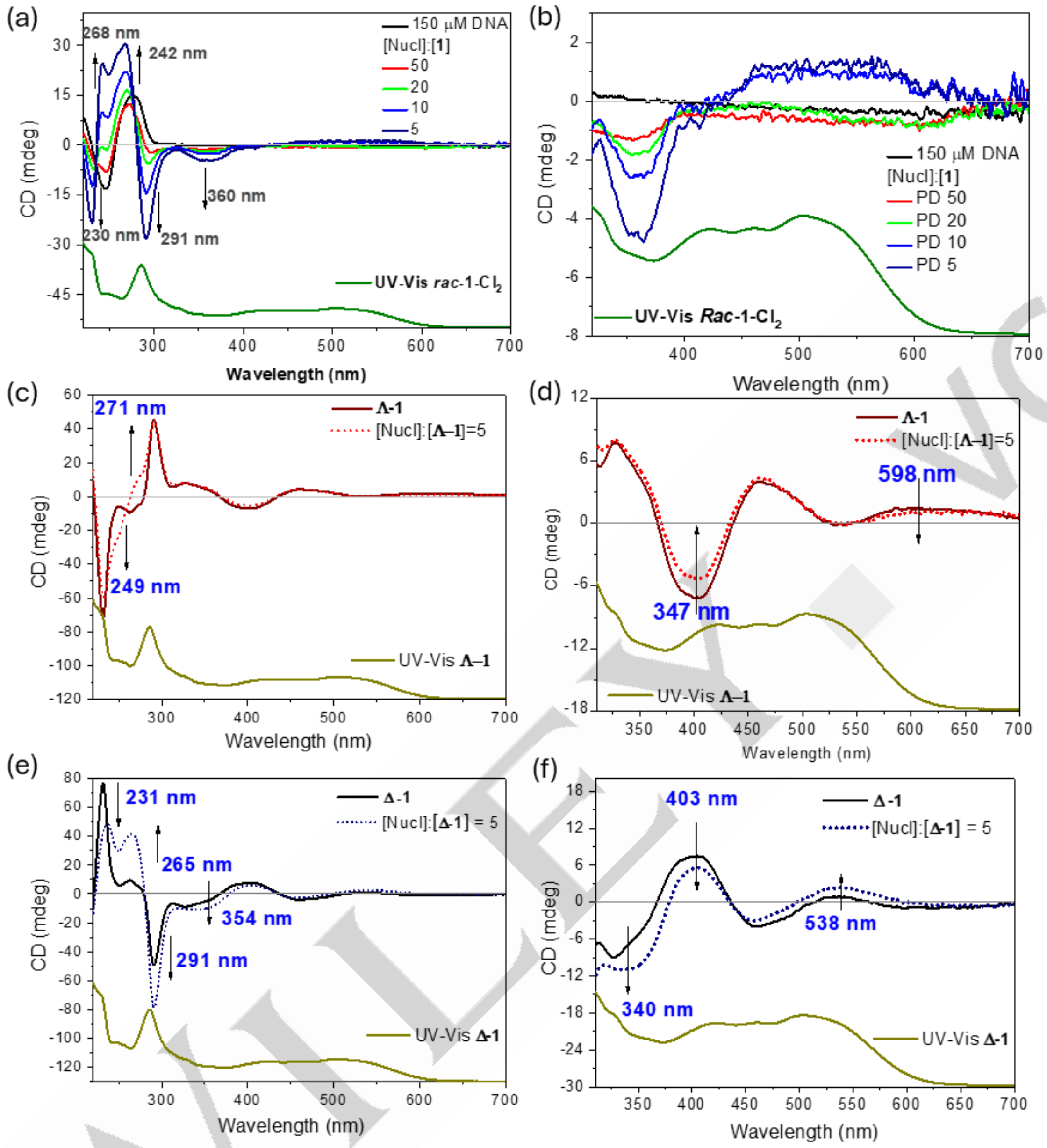

Figure S12. Circular dichroism spectra of (a,b) [*rac*-**1**](Cl)$_2$, (c,d) [Λ-**1**]Cl$_2$ and (e,f) [Δ-**1**](Cl)$_2$ (30 μM) in 50 mM potassium phosphate buffered aqueous solution in the absence and presence of st-DNA (150 μM, [Nuc]:[**1**]= 5).

WILEY-VCH

Thermal Denaturation Studies

Table S12. Melting temperature values for thermal denaturation of st-DNA (75 µM bp) in 1 mM aqueous potassium phosphate buffered solution with 2 mM NaCl at pH 7.4, in the absence and presence of [Λ-**1**]$(Cl)_2$ and [Δ-**1**]$(Cl)_2$ at a [Nucl]:[**1**] ratio of 20.

***T*m [°C]**	**Derivative Λ**	**Derivative Δ**
1	60.0	61.2
2	59.8	62.0
3	60.4	62.6
Mean	60.1 ± 0.3	61.9 ± 0.6
ΔT_m (°C)	2.1 ± 0.2	4.0 ± 0.6

References

[1] E. M. Kober, J. V. Caspar, B. P. Sullivan, T. J. Meyer,Synthetic routes to new polypyridyl complexes of osmium(II) *Inorg. Chem.* **1988**, *27*, 4587-4598.
[2] N. Alatrash, F. H. Issa, N. S. Bawazir, S. J. West, K. E. Van Manen-Brush, C. P. Shelor, A. S. Dayoub, K. A. Myers, C. Janetopoulos, E. A. Lewis, F. M. MacDonnell,Disruption of microtubule function in cultured human cells by a cytotoxic ruthenium(ii) polypyridyl complex *Chem. Sci.* **2020**, *11*, 264-275.
[3] G. M. Sheldrick,SHELXT– Integrated space-group and crystal-structure determination *Acta Crystallogr. A.* **2015**, *71*, 3-8.
[4] O. V. Dolomanov, L. J. Bourhis, R. J. Gildea, J. A. K. Howard, H. Puschmann,OLEX2: a complete structure solution, refinement and analysis program *J. Appl. Crystallogr.* **2009**, *42*, 339-341.
[5] A. Spek,Single-crystal structure validation with the program PLATON *J. Appl. Crystallogr.* **2003**, *36*, 7-13.
[6] G. Lucena-Aguilar, A. M. Sánchez-López, C. Barberán-Aceituno, J. A. Carrillo-Ávila, J. A. López-Guerrero, R. Aguilar-Quesada,DNA Source Selection for Downstream Applications Based on DNA Quality Indicators Analysis *BIO.* **2016**, *14*, 264-270.
[7] M. T. Carter, M. Rodriguez, A. J. Bard,Voltammetric studies of the interaction of metal chelates with DNA. 2. Tris-chelated complexes of cobalt(III) and iron(II) with 1,10-phenanthroline and 2,2'-bipyridine *J. Am. Chem. Soc* **1989**, *111*, 8901-8911.
[8] C. Adamo, D. Jacquemin,The calculations of excited-state properties with Time-Dependent Density Functional Theory *Chem. Soc. Rev.* **2013**, *42*, 845-856.
[9] T. Yanai, D. P. Tew, N. C. Handy,A new hybrid exchange–correlation functional using the Coulomb-attenuating method (CAM-B3LYP) *Chem. Phys. Lett.* **2004**, *393*, 51-57.
[10] S. Grimme, S. Ehrlich, L. Goerigk,Effect of the damping function in dispersion corrected density functional theory *J. Comput. Chem.* **2011**, *32*, 1456-1465.
[11] F. Weigend, R. Ahlrichs,Balanced basis sets of split valence, triple zeta valence and quadruple zeta valence quality for H to Rn: Design and assessment of accuracy *PCCP.* **2005**, *7*, 3297-3305.
[12] L. E. Roy, P. J. Hay, R. L. Martin,Revised Basis Sets for the LANL Effective Core Potentials *J. Chem. Theory Comput.* **2008**, *4*, 1029-1031.
[13] S. I. Gorelsky, A. B. P. Lever,Electronic structure and spectra of ruthenium diimine complexes by density functional theory and INDO/S. Comparison of the two methods *Journal of Organometallic Chemistry* **2001**, *635*, 187-196.
[14] C. Legault, *CYLview20 - Quick Guide*, **2020**.
[15] M. J. Frisch, G. W. Trucks, H. B. Schlegel, G. E. Scuseria, M. A. Robb, J. R. Cheeseman, G. Scalmani, V. Barone, G. A. Petersson, H. Nakatsuji, X. Li, M. Caricato, A. V. Marenich, J. Bloino, B. G. Janesko, R. Gomperts, B. Mennucci, H. P. Hratchian, J. V. Ortiz, A. F. Izmaylov, J. L. Sonnenberg, Williams, F. Ding, F. Lipparini, F. Egidi, J. Goings, B. Peng, A. Petrone, T. Henderson, D. Ranasinghe, V. G. Zakrzewski, J. Gao, N. Rega, G. Zheng, W. Liang, M. Hada, M. Ehara, K. Toyota, R. Fukuda, J. Hasegawa, M. Ishida, T. Nakajima, Y. Honda, O. Kitao, H. Nakai, T. Vreven, K. Throssell, J. A. Montgomery Jr., J. E. Peralta, F. Ogliaro, M. J. Bearpark, J. J. Heyd, E. N. Brothers, K. N. Kudin, V. N. Staroverov, T. A. Keith, R. Kobayashi, J. Normand, K. Raghavachari, A. P. Rendell, J. C. Burant, S. S. Iyengar, J. Tomasi, M. Cossi, J. M. Millam, M. Klene, C. Adamo, R. Cammi, J. W. Ochterski, R. L. Martin, K. Morokuma, O. Farkas, J. B. Foresman, D. J. Fox, Wallingford, CT, **2016**.

WILEY-VCH
